# Supplementary material for: BLINK: a package for the next level of genome-wide association studies with both individuals and markers in the millions
Source: Gigascience. 2018 Dec 11;8(2):giy154. doi: 10.1093/gigascience/giy154 (PMC6365300; doi:10.1093/gigascience/giy154)
Supplement: Supplemental Files [file giy154_supplemental_files.zip › BLINK_main_MH.docx]

**BLINK: A Package for the Next Level of Genome-Wide Association Studies with Both Individuals and Markers in the Millions**

Meng Huang^1^, Xiao-lei Liu^2^, Yao Zhou^1^, Ryan M. Summers^3^ and Zhi-wu Zhang^1*^

^1^Department of Crop and Soil Sciences, Washington State University, 1170 NE stadium Way Pullman, Washington State 99164-6420, United States of America

^2^Key Laboratory of Agricultural Animal Genetics, Breeding and Reproduction, Ministry of Education, College of Animal Science and Technology, Huazhong Agricultural University, 1 Shizishan Street, Wuhan, Hubei 430070, China

^3^School of Electrical Engineering and Computer Science, Washington State University, 355 NE Spokane Street, Pullman, Washington State 99164-2752, United States of America

*Correspondence: ZZ (zhiwu.zhang@wsu.edu)

**Abstract**

Big datasets, accumulated from biomedical and agronomic studies, provide the potential to identify genes controlling complex human diseases and agriculturally important traits through genome-wide association studies (GWAS). However, big datasets also lead to extreme computational challenges, especially when sophisticated statistical models are employed to simultaneously reduce false positives and false negatives. The newly developed Fixed and random model Circulating Probability Unification (FarmCPU) method uses a bin method under the assumption that Quantitative Trait Nucleotides (QTNs) are evenly distributed throughout the genome. The estimated QTNs are used to separate a mixed linear model into a computationally efficient fixed effect model (FEM) and a computationally expensive random effect model (REM), which are then used iteratively. To completely eliminate the computationally expensive REM, we replaced REM with FEM by using Bayesian Information Criteria. To eliminate the requirement that QTNs be evenly distributed throughout the genome, we replaced the bin method with linkage disequilibrium information. The new method is called Bayesian-information and Linkage-disequilibrium Iteratively Nested Keyway (BLINK). Both real and simulated data analyses demonstrated that BLINK improves statistical power compared to FarmCPU, in addition to remarkably reducing computing time. Now, a dataset with one-half million markers and one million individuals can be analyzed within five hours, instead of one week using FarmCPU.

**Keywords**: GWAS; big datasets; complex traits; computationally efficient model

**Introduction**

Biomedical innovations have outpaced computing innovations since the completion of the human genome project[1,2]. Genome-wide association studies (GWAS) have identified many genetic loci presumed to control some human diseases and agriculturally important traits[3–5]. However, a substantial proportion of these discoveries were false positives, attributed to a failure to consider population structure and cryptic relationships among individuals in the analyses[6–8]. Incorporating population structure and cryptic relationships as covariates dramatically reduce false positives, but also cause false negatives and computational burdens[9–11].

Population structure is typically incorporated as a fixed effect in the General Linear Model (GLM), which is computationally efficient. Initially, population structure was derived as the proportions of individuals belonging to sub-populations[12,13]. Several alternatives for defining population structure, such as principal component analysis (PCA)[14,15], were developed to further improve computational efficiency. The population structure and PCA methods are efficient to incorporate sub-population effects, but not capable to model the cryptic relationship among individuals within sub-populations. Incorporating both effects of sup-populations and cryptic relationship among individuals further reduce false positives and increase statistical power[9]. Cryptic relationships can be incorporated in two ways. One way is to include all genetic markers as random effects. Some of these markers will capture the effects of Quantitative Trait Nucleotides (QTNs) through Linkage Disequilibrium (LD)[16–18]. The other way is to first derive kinship among individuals, using all genetic markers. The kinship is subsequently used to define the variance structure of individual effects as random effects. In the latter, both population structure and kinship can be incorporated into a fixed effect and random effect Mixed Linear Model (MLM)[9]. However, the computation of the MLM is intensive. Thus, multiple methods have been developed to reduce the computing times of MLM.

The first milestone that reduced this computational burden was the development of the Efficient Mixed Model Association (EMMA)[19]. Prior to EMMA methods, Maximum Likelihood (ML) or REstricted Maximum Likelihood (REML) performed a two-dimensional optimization of the genetic variance and the residual variance using methods such as Expectation and Maximization (EM). By using EMMA, ML or REML is a function of the ratio between genetic variance and residual variance. By reducing the optimization from two dimensions (genetic variance and residual variance) to one dimension (genetic-to-residual variance ratio), computing speed dramatically improves.

The second milestone was the use of empirical Bayesian estimation of population parameters such as genetic and residual variances or their ratio. This method is based on the assumption that each testing marker contributes only a small proportion of total genetic variance. Thus, population parameters for the testing markers can be approximated by the estimates from a reduced model without fitting each marker[20,21]. Developed independently by two different groups, this algorithm has two names, Population Parameters Previously Determined (P3D)[21] and EMMA eXpedited (EMMAX)[20]. Inspired by EMMA, EMMAx, and P3D, an exact algorithm, Genome-wide Efficient Mixed-Model Association (GEMMA)[22], was developed to derive estimates of population parameters for each testing marker with the same computing speed as P3D and EMMAX.

The third milestone was the Compressed MLM (CMLM) that clusters individuals into groups based on kinship[21]. The computing time complexity of MLM is the cubic power of the number of equations. Clustering individuals into groups reduces the number of equations from the number of individuals to the number of groups. Consequently, computing time is dramatically reduced in CMLM. Clustering individuals into groups is performed in a reduced model without fitting testing markers. The optimized grouping is used to test markers one at a time. The computing advantage of CMLM is greater for datasets with larger numbers of markers.

The fourth milestone was a method called Factored Spectrally Transformed Linear Mixed Models (FaST-LMM)[23], which uses a rank-reduced kinship. Rather than using all available markers, a subset of genetic markers—less than the number of individuals in the sample—is used to create the rank-reduced kinship. Furthermore, FaST-LMM directly uses this subset of markers to define the relationship among individuals for ML or REML optimization without first calculating kinship. As a result, computing time is linear to the size of the marker subset and independent of sample size.

The fifth milestone was GRAMMAR-Gamma[24], a method that splits the association analysis into two steps. The first step uses MLM to derive the residuals. The second step tests the residuals as transformed traits in a fixed effect model and applies a correction factor to test statistical values. The computing complexity of the second step is completely linear to both number of individuals and number of markers.

With the exception of CMLM, the primary aim of the above milestones was to improve computing speed. The statistical power of each of these milestones remains similar to the conventional MLM[9] because the same or similar kinship is used regardless of the traits being analyzed. CMLM, on the other hand, represented the first adjustment of kinship to improve statistical power[21]. In CMLM, genetic effects of individuals in the conventional MLM are replaced by the genetic effects of their corresponding kinship groups. That is, kinship among individuals is replaced by kinship among groups. Furthermore, the adjustment on kinship is optimized for the particular traits being studied. For example, the kinship with the best ML or REML is used for testing markers. Other optimizations were also developed to define the minimum and maximum group kinship, in addition to average kinship[25].

The second adjustment to improve statistical power employs kinship that is not only specific for traits, but also specific for testing markers[26,27]. Kinship is built by using only the markers that are associated with a trait. Because multiple associated markers can be genetically linked, a bin procedure was developed to remove this redundancy. The procedure was named the Settlement of MLM Under Progressively Exclusive Relationship (SUPER), which ensures that, at most, only one associated marker is selected from each bin[27]. Furthermore, the kinship changes according to testing markers to eliminate the confounding between kinship and testing markers. The trait-associated markers are excluded from the kinship calculation if they are also associated with the testing markers. This association is determined by linkage disequilibrium (LD) in SUPER[27]. In FaST-LMM-SELECT, the associated markers are removed if they are on the same fragment (within 1Mb) as the testing markers[26].

The third adjustment, known as the multi-locus mixed-model (MLMM) approach, applies elimination of the kinship[28]. In addition to random individual effects, this adjustment also fits multiple associated markers in the MLM to split the variance explained by kinship in a stepwise regression fashion. The forward stepwise regression stops when the variance explained by the kinship is near zero. The associated markers are re-selected through backward regression. The final set of associated markers, named pseudo QTNs, are fitted as covariates to test the remaining markers with a Fixed Effect Model (FEM).

Recently, a fourth adjustment was developed, called the Fixed and random model Circulating Probability Unification (FarmCPU)[29]. FarmCPU uses REML to replace the criterion that the variance explained by kinship is near zero, which can only be arbitrarily determined. FarmCPU also adapted the bin approach from SUPER to select pseudo QTNs. The whole genome is equally divided into a certain number of bins, and only one significant marker with the smallest P value from each bin is selected as the candidate pseudo QTN. These candidate pseudo QTNs are determined by a Random Effect Model (REM). The candidate pseudo QTNs, from the various bins with different widths, are first ranked by P value. Then, the best combinations between the different bins and the number of candidate pseudo QTNs are determined by REM. Finally, the two types of models (FEM and REM) are performed iteratively until no change occurs in the selection of pseudo QTNs.

Despite these valuable advancements, more innovative computing tools and analysis methods are needed. For example, although FarmCPU boosts statistical power in GWAS, its REM process remains computationally demanding. Additionally, the bin approach from SUPER requires that all QTNs be evenly distributed throughout the genome, which is rarely true. Furthermore, only one QTN can be selected as a covariate even if multiple QTNs are located in the same bin, which limits statistical power. Thus, a critical need still exists for a method that can increase both computing efficiency and statistical power.

**Results**

Herein, we developed a new statistical method that was inspired by this critical need and builds upon our previous method, FarmCPU. In the new method, we use 1) Bayesian Information Criteria (BIC) in a FEM to replace REML in the REM, and 2) linkage disequilibrium information to replace the bin method. As a result, we have completely eliminated the computationally expensive REM and the requirement that QTNs be evenly distributed throughout the genome (Fig. 1). We named the new method Bayesian-information and Linkage-disequilibrium Iteratively Nested Keyway (BLINK). The BLINK procedure is further detailed in the online methods section.

We implemented the BLINK algorithm in two statistical software packages; one was written in R and the other in C. The R package was designed for the popularity of R users. The C package was designed for computational efficiency. We named the two packages BLINK-R and BLINK-C, respectively. The results from the two packages are identical (Fig. S15). The difference is that BLINK-C is much faster than BLINK-R. Because most of the analyses were conducted by BLINK-C in this study, hereafter, we simplified BLINK-C to BLINK. We compared BLINK's computing speed and statistical power with two complementary software packages, PLINK[30] and FarmCPU[29]. PLINK was written in C and implements the GLM method, which has the minimum theoretical computing time complexity. FarmCPU was written in R and implements the FarmCPU algorithm, which is superior to GLM with respect to statistical power.

Comparisons of statistical power were based on false positives, true positives, and statistical power at different levels of false discovery rate (FDR) and Type I error (Fig. 2). To retain the real population structure (Fig. S1), we used phenotypes simulated from real genotypes that covered a wide range of species, including human, one crop (maize), one livestock (pig), and two model species (*Arabidopsis* and mouse). Additionally, we conducted association studies on real phenotypes to assess the flowering time trait in maize (Fig. 3). Enrichment was performed in a different study to compare BLINK and FarmCPU (Fig. 4). Real phenotypes were also analyzed to cover a wide range of species (Fig. S 4-7), including human, livestock (pig), and two model species (*Arabidopsis* and mouse). Finally, real genotype and phenotype data were duplicated to synthetically create a big dataset to compare observed computing times of BLINK-C, BLINK-R, PLINK, and FarmCPU (Fig. 5).

***Receiver operating characteristic (ROC) curve analysis***

Using real genotypes from all five species, we simulated the QTNs controlling the phenotypes in two scenarios. The first scenario was a situation that rarely, if ever, occurs in practice—all QTNs were randomly located on the chromosomes without being clustered. We called it the "synthetic" scenario. The second scenario was a situation closer to reality—QTNs were clustered on chromosomes. Every two QTNs were located within 10 Kb of each other. We called it the "real" scenario. For each scenario, we examined statistical power under different levels of FDR and Type I error. FDR was defined as the proportion of false positives among the total number of true positives. Type I error was derived from the empirical null P-value distribution of all non-QTN-bins. The relationship between statistical power and FDR or Type I error is described by the receiver operating characteristic (ROC) curves (Fig 2 and S 8-12). The method with a larger area under curve (AUC) is preferred over the method with a smaller AUC. BLINK had a larger AUC than FarmCPU, BOLT-LMM[31], and PLINK for both power versus FDR and power versus Type I error; PLINK and BOLT-LMM had a smaller AUC than FarmCPU and BLINK for both comparisons. This situation held true across all five species.

The model selection criteria was compared among BIC (Bayesian Information Criteria), AIC (Akaike Information Criterion)[32], and EBIC (Extended BIC)[33] in all the five species examined. BIC outperformed the other two model selection criteria (Fig S13). The determination of two markers in LD was based on the absolute values of their Person correlation coefficient. BLINK chose 0.7 as the default value based on the comparisons of statistical power under different FDR (Fig S14).

***Associations and enrichment on real phenotypes***

We conducted GWAS on flowering time in maize using the four methods (BLINK, FarmCPU, BOLT-LMM and PLINK). PLINK exhibited strongly inflated P values (Fig. 3). For example, of the 397,323 SNPs in maize, PLINK identified 48,194 (12%) SNPs with P values smaller than the Bonferroni threshold. This result was consistent with the result on the 282 maize association panel, where incorporating the population structure matrix (Q) did not control inflation as well as the Q + K (kinship) model[9]. Including more covariates such as PCs in PLINK reduced the number of significant SNPs (**S17 Figure**), however, this might reduce the true positives as documented in previous study on with increasing number of PCs with GLM[29].

In addition to population structure, the cryptic relationships among individuals also contributed to the inflation of P values. One way to solve the problem is to remove the related individuals. With kinship cutoff of 0.5, the number of individuals was reduced from original 2,279 individuals to 1,218 individuals. This pruning strategy not only reduce the sample size and statistical power consequently, but, also retain substantial inflation of P values. There were still 211 SNPs over 1% threshold after Bonferroni multiple test correction. BOLT-LMM, FarmCPU and BLINK controlled inflation well. Results from BLINK and FarmCPU indicated that more than 99.9% of SNPs were not associated with flowering time after the adjustments by the associated SNPs. BLINK, BOLT-LMM and FarmCPU had much better control on inflation of P values across the genome than PLINK.

Notably, with about the same control on inflation of P values, BLINK identified more associated SNPs than FarmCPU. FarmCPU identified 14 SNPs that passed the Bonferroni multiple test threshold (α = 0.01). In contrast, BLINK not only revealed 9 of these 14 SNPs, but also identified an additional 40 loci that passed the Bonferroni threshold. The significant SNPs identified by BLINK included the SNPs that are 2 Kb from ZmCCT, 441 kb from ZCN8, and 568 kb from Vgt1—the three genes that have been previously cloned (Fig. 3). These three genes were also the QTLs detected in the Nested Association (NAM) population[34]. FarmCPU also identified the SNP that is 2 Kb away from ZmCCT, but not the other two SNPs near ZCN8 and Vgt1. The SNPs detected by FarmCPU were further away from ZCN8 and VGT1, compared to the ones detected by BLINK. Both FarmCPU and BLINK detected some NAM QTLs, including the 16^th^ and 27^th^ NAM QTLs.

Although NAM and the population we used in this study were different, they are strongly connected because the parents of NAM are part of the population we used. This relationship could partially explain the overlaps, and we were interested to find overlaps between the different populations. Recently, a distinctly bigger population, with 4,471 landraces, was used to dissect the genetic architecture of maize flowering time through GWAS. To distinguish genes for local environmental adaptation, GWAS was conducted in conjunction with controlled field experiments through a newly developed experimental design called F-one association mapping (FOAM)[35]. FOAM sampled individuals and crossed them with a small number of common parents to derive F1 families. GWAS was then used to evaluate multiple trial F1 progeny and identified 1,003 genes associated with flowering time.

The 9 genetic loci identified by both FarmCPU and BLINK, and the 40 genetic loci uniquely identified by BLINK, were significantly enriched on the 1,003 flowering time genes identified by FOAM. The flowering time gene regions were defined as 50 Kb upstream and downstream of the 1,003 genes. These regions occupied about 3% of the maize genome. The strength of the enrichment was indicated by the difference between the observed number of genetic loci hitting the FOAM flowering time gene regions and the expected number under the null hypothesis that genetic loci were selected randomly. The detailed derivations of the expected null distributions are illustrated in the Method section.

Among the nine associated loci identified by BLINK, three were located in the flowering time gene regions. The chance to have four or more overlaps was above 1% if the nine loci common to both FarmCPU and BLINK were randomly selected. The five genetic loci unique to FarmCPU were not enriched; but, the 40 genetic loci unique to BLINK were significantly enriched. Among these 40 associated loci, eight were located on the flowering time gene regions. The chance to have eight or more overlaps was below 5% if these 40 loci were selected randomly (Fig. 4).

***Theoretical computing times***

In PLINK, association analysis of *M* markers with *c* covariates on a sample with *N* individuals takes a total computing time of c^2^MN. The quadratic term comes from the inverse of the left-hand side of the coefficient matrix. Both FarmCPU and BLINK add at most *t* pseudo QTNs as additional covariates to simultaneously control false positives and reduce false negatives. FarmCPU performs the model selection of these pseudo QTNs with a REML procedure in the REM. The REM is solved to optimize bin size (*b*) and the number of pseudo QTNs and to optimize the genetic-to-residual variance ratio with *p* iterations. FarmCPU's computing time is tbp(c+t)^2^N for model selection and (c+t)^2^MN for association tests; total computing time is (M+tbp)(c+t)^2^N.

BLINK replaced REM with FEM for the model selection of *t* pseudo QTNs. Consequently, the iterations are eliminated for optimizing the genetic-to-residual variance ratio. BLINK has a computing time of (c+t)^2^N for selecting pseudo QTNs. The total computing time for BLINK is (M+t)(c+t)^2^)N+(c+t)^2^N. The number of common covariates (*c*), pseudo QTNs (*t*), bin sizes (*b*), and iterations (*p*) are much smaller than both M and N. These scalars remain constant regardless of M and N sizes. Therefore, the computing time complexity is MN with respect to big O for all three methods (PLINK, FarmCPU, and BLINK) (Table 1).

***Observed computing times***

We compared the two BLINK packages' (C and R) computing times for analyzing big datasets with PLINK[30] and FarmCPU[29] (Fig. 5). The datasets were synthetically created by randomly duplicating 8,800 human individuals genotyped with one-half million SNPs. The largest synthetic dataset contained one million individuals. FarmCPU took about 4 hours to complete the analysis on a dataset with about 20,000 individuals. During that same timeframe, BLINK-R completed the analysis on a dataset with about 50,000 individuals. PLINK 1.9 analyzed the largest dataset (one million individuals) in about 7 hours, while BLINK-C only needed 3 hours. BLINK-R was about three times faster than FarmCPU. BLINK-C was about 20 times faster than BLINK-R. BLINK-C was about two times faster than PLINK 1.9. These results suggest that platforms and coding played an important role in computation efficiency for implementing the same algorithms.

Among the four packages compared above, BLINK-C can fully use modern computer architecture with multiple central processing unit (CPU) cores for parallelization. We further examined the efficiency of BLINK-C on multiple-core computer systems. We tested BLINK-C on computers with core numbers ranging from 2 to 12 under Linux and Mac (Table S2). Results showed that the total computing time decreased linearly with the number of cores (Fig. 5). For the dataset with about one million individuals and one-half million SNPs, a Mac Pro with 12 cores completed the analysis in just 30 minutes instead of 3 hours with a single core.

**Discussion**

Inspired by the critical need for computational efficiency and statistical power in big dataset analysis and by the recently developed GWAS method, FarmCPU, we developed a faster and more powerful method. By substituting REML in FarmCPU's REM with BIC in a FEM and by replacing the bin approach with LD, we achieved optimization in one dimension (number of pseudo QTNs) instead of two dimensions (number of pseudo QTNs and bin size). The optimization of the genetic-to-residual variance ratio was also eliminated by substituting REML with BIC, which directly solves residual variance without iterations. These improvements not only reduced computing time, but also simultaneously reduced false positives and false negatives.

***Substitution of REML with BIC***

In both FarmCPU and BLINK models, markers are tested one at a time, with pseudo QTNs added as covariates to control false positives and reduce false negatives. FarmCPU selects these pseudo QTNs by using REM. Pseudo QTNs are used to derive kinship among individuals. The model chooses a set of pseudo QTNs to derive a kinship that provides the maximum likelihood[29]. Because FarmCPU does not gain extra parameters as more pseudo QTNs are included, the likelihood is not penalized for having more pseudo QTNs. In contrast, BLINK chooses pseudo QTNs using FEM. The more pseudo QTNs included, the greater the likelihood. Therefore, a penalty, such as BIC, on the number of parameters is necessary to identify the set of pseudo QTNs that best controls false positives and reduces false negatives. Both simulated data and real data demonstrated that BIC penalization worked well. By jointly using BIC and substituting for the bin approach, BLINK's FEM performed even better than REML in FarmCPU.

***Robustness with genetic architecture***

The FarmCPU method uses bins as pseudo QTNs, according to the SUPER GWAS method[27,29]. Both the number of bins (pseudo QTNs) and size of bins must be optimized together, in addition to optimizing the genetic-to-residual variance ratio. BLINK performs optimization in only one dimension (number of pseudo QTNs). A pseudo QTN represents a single SNP, not a bin. Multiple pseudo QTNs are acceptable regardless of proximity on the genome, unless they are in LD. In contrast, with FarmCPU, only one pseudo QTN can be selected if multiple pseudo QTNs are close enough to fall into the same bin. In practice, real QTNs are often clustered, rather than evenly distributed; thus, BLINK is more robust than FarmCPU.

***Optimization of model selection***

The selection of pseudo QTNs is influenced by the threshold that determines whether a pair of SNPs are highly correlated. The current default setting used in BLINK-C and BLINK-R is 70% (Pearson correlation coefficient). We evaluated all of BLINK's default settings in all five populations (Fig. S 13-14). Although these default settings work well, different criteria and/or methods may further improve optimization for specific species and/or datasets—a topic that remains open to future research.

Nevertheless, BLINK produced fewer false positives and identified more true positives than the most recently developed GWAS method, FarmCPU. BLINK outperformed FarmCPU[29] and PLINK[30] relative to both statistical power versus FDR and statistical power versus Type I error. The association analyses with BLINK identified more genetic loci, including loci previously validated by other studies, than PLINK or FarmCPU. Although BLINK has the same computing time complexity as PLINK and FarmCPU, BLINK-C was not only faster than FarmCPU, but also faster than PLINK 1.9[36]. BLINK-C can analyze an extremely big dataset—one million individuals and one-half million markers—in 3 hours with a single core, or in 30 minutes with 12 cores.

**Materials and methods**

***BLINK procedure***

The BLINK method conducts two Fixed Effect Models (FEMs) and one filtering process, which selects a set of pseudo QTNs that are not in LD with each other as covariates. The entire sequence runs repeatedly until all genetic markers are tested and the selection of pseudo QTNs is optimized. The first FEM tests *M* genetic markers, one at a time. Pseudo QTNs are included as covariates to simultaneously control false positives and reduce false negatives. Specifically, the first FEM can be written as follows:

y_i_ = S^*^_i1_b_1_ + S^*^_i2_b_2_ + ... + S^*^_ik_b_k_ + S_ij_d_j_ + e_i_ (1)

where y_i_ is the observation on the i^th^ individual; S_i1_, S_i2_,…, S_ik_ are the genotypes of k pseudo QTNs, initiated as an empty set; b_1_, b_2_, …, b_k_ are the corresponding effects of the pseudo QTNs; S_ij_ is the genotype of the i^th^ individual and j^th^ genetic marker; d_j_ is the corresponding effect of the j^th^ genetic marker; and e_i_ is the residual having a distribution with a mean of zero and a variance of$\sigma_{e}^{2}$. The primary goal of the first FEM is to calculate the P values for all *M* testing markers.

The second FEM is employed to optimize the selection of pseudo QTNs. Specifically, the second FEM can be written as follows:

y_i_ = S^*^_i1_b_1_ + S^*^_i2_b_2_ + ... + S^*^_ik_b_k_ + e_i_ (2)

Equations (1) and (2) differ in two ways. First, the testing marker term in the first FEM is removed from the second FEM; therefore, no testing marker P values are output in equation (2). Second, the number of covariate pseudo QTNs are varied in the second FEM to select the optimum set of the first k out of t pseudo QTNs. The optimization is performed using Bayesian Information Criteria (BIC), which is twice the negative log likelihood plus the penalty on number of parameters, as follows:

BIC = -2LL + 2kLn(n) (3)

where LL is the Log Likelihood, k is the number of pseudo QTNs, Ln is the natural Log, and n is the number of individuals. The available pseudo QTNs, t, are sorted with the most significant at beginning and the least significant at end. The first k pseudo QTNs are selected for examination, with k varied from 1 to t.

All markers in equation (1) are candidates for pseudo QTNs in equation (2). These markers are filtered with two criteria: P value and correlation. All markers are sorted first and then filtered out if their P values are larger than a threshold (Bonferroni correction, α = 0.01). Of the m SNPs remaining, if their correlation, r (Pearson correlation), with the first SNP (S_1_^*^) is larger than a threshold (0.7), they are also removed. This process is repeated to select S_2_^*^, S_3_^*^,…, until the last SNP, S_t_^*^, is selected (Fig 1).

Because the t remaining markers are sorted and not highly correlated each other, the first set of k markers is more critical than the second set of k markers. We fit the first k markers in equation (2) and vary k until all possibilities are examined. The set of k markers with the best BIC is used as the set of pseudo QTNs in equation (1). This process is iterated until the pseudo QTNs remain the same. We named this alternative solution as Bayesian-information and Linkage-disequilibrium Iteratively Nested Keyway (BLINK) method**.**

***Genotype and phenotype data***

We used the exact same datasets we used in our previous publication for the FarmCPU method. These datasets covered five species including *Arabidopsis thaliana*[10], human[5], maize[37], mouse[38], and pig[39]. Markers with a Minor Allele Frequency (MAF) of 5% or below were filtered out from the original datasets. The number of individuals and markers and traits are summarized in Table S1. The principal components were calculated by PLINK using all the SNPs. The Manhattan plots of GWAS results were drawn using GAPIT[40,41].

In the maize dataset[37], all samples were inbred lines from the USDA Plant Introduction Station in Ames, Iowa. A total of 2,279 inbred lines comprised this dataset, each line with 681,258 SNPs. The real phenotype of all dataset samples was flowering time, which was measured as days to silking. Both genotypes (ZeaGBSv1.0) and phenotypes (USDA Ames inbred collection phenotypes) were downloaded from Panzea (URL of genotype: http://cbsusrv04.tc.cornell.edu/users/panzea/filegateway.aspx?category=Genotypes; URL of phenotype: https://www.panzea.org/phenotypes).

The human dataset was obtained from dbGaP[5]. The name of this dataset is “East Asian lung cancer dataset” (ID # phs000716.v1.p1). Respecting the privacy and intentions of research participants, this dataset is only available under the permission of NIH (National Institutes of Health) and Intramural NCI (National Cancer Institute). The dataset includes a total of 8,807 samples, which were collected from China, Korea, and Japan. These samples, each with 629,968 SNPs, were involved in our computing efficiency tests. (URL: http://www.ncbi.nlm.nih.gov/projects/gap/cgi-bin/study.cgi?study_id=phs000716.v1.p1).

We used two datasets of *Arabidopsis* *thaliana*[10]. The larger dataset, containing 1,179 individuals that were genotyped with 214,545 SNPs, was used for our power and FDR simulation tests ([URL: http://archive.gramene.org/diversity/download_data.html](file:///Users/Zhiwu/Dropbox/Current/paper/BLINK/Manuscript/GIGA%20Science/V3/%20http:/archive.gramene.org/diversity/download_data.html); Dataset: 2010 project 250K SNP chip genotypes v3.04). The smaller dataset with 199 individuals was used for the real trait GWAS tests (Dataset: Atwell et. Al, Nature June 2010; Phenotype: flowering time at 16°C).

The mouse dataset[38], containing 1,940 samples (1,000 males and 940 females) with 12,226 SNPs, came from a heterogeneous stock mice population owned by the Welcome Trust Centre for Human Genetics (University of Oxford, UK). We used the growth slope phenotype data in our real trait association tests.

The pig genotype dataset[39] included 820 individuals (412 Large White and 408 crosses from Large White and Landrace) with 64,212 SNPs. We used the last rib back-fat thickness phenotype data in our real trait association tests.

The population structure of these five test datasets was identified using the first two PCs in Fig. S1. Phenotype distributions were illustrated as scatter plots, histogram plots, and box plots in Fig. S2.

***Synthetic data and computing speed evaluation***

The human dataset was synthetically duplicated to evaluate computing efficiency on large-scale datasets. The human dataset contained about one-half million (629,968) SNPs and 8,807 individuals. Individuals were randomly selected to amplify sample size to 10,000, 20,000, 50,000, 100,000, 200,000, 500,000, and 1,000,000. The number of SNPs remained the same, at approximately one-half million. The function of creating synthetic datasets has been added into BLINK to allow a user to generate the synthetic dataset. The R demo code is illustrated in GitHub (https://github.com/Menggg/BLINK/blob/master/synthetic_genotype_data.R) to explain how to use the BLINK demo data to generate the synthetic dataset.

Computing speed comparisons between BLINK, FarmCPU, and PLINK 1.9 were conducted on the same computer. Parallel computing performance was tested on computers with different Operating Systems (OS) and machine configurations (Table S2).

***Simulated phenotypes***

The real genotypes of the five species were used to simulate phenotypes to examine statistical power under different levels of Type I error and FDR. The simulated phenotypes had a heritability of 75%, controlled by a variable number of QTNs that were sampled from all real SNPs. Two scenarios, with and without restriction, were applied to the sampling of SNPs. The restriction was that a QTN must be within a 300 Kb distance of another QTN. The QTNs had effects that followed a normal distribution. These QTNs were summed together as the total additive genetic effect for each individual, according to its real genotype. The variance of additive genetic effect was calculated across all individuals. A normally distributed residual effect was assigned to each individual. The variance of the residual effect was assigned accordingly, so that the proportion of additive genetic variance equaled heritability. Genomes were divided into different bin sizes (1 bp, 1 KB, and 100 KB). Bins were classified as QTN bins if they contained at least one QTN, otherwise, as non-QTN bins. The P value of a bin was represented by the most significant single nucleotide polymorphism (SNP) in the bin. Statistical power was defined as the proportion of QTNs detected for each different level of false discovery rate (FDR). Type I error was derived from the empirical null distribution of non-QTN bins.

***Power, Type I error, and FDR***

The numbers of false and true positives were counted based on bins, as described in our previous study[29]. Bin size was varied, ranging from a single base pair to one mega base pairs. We reported the results from using different bin sizes (1 bp, 1 KB, and 100 KB). The P value of a bin was represented by its most significant SNP. A bin was considered a QTN-bin if it contained at least one QTN, otherwise, a non-QTN-bin. A non-QTN-bin with a P value that passed a threshold was counted as a false positive bin. A QTN-bin with a P value that passed the same threshold was counted as a true positive bin. The proportion of QTNs identified under different thresholds was calculated as statistical power. For all levels of statistical power, the proportion of non-QTN-bins was calculated as FDR. Type I error was derived from the empirical null distribution of all non-QTN bins. Furthermore, receiver operating characteristic (ROC) curves were used to compare statistical power under different levels of FDR and Type I error**.** The area under the curve (AUC) was calculated with a starting point of zero and an ending point of one for FDR or Type I error.

**Availability of software and source code**

Project name: BLINK

Project home page: <http://zzlab.net/blink>

Operating systems: Mac OS and Linux

Programing Language: C, R and OpenCL

License: GNU General Public License version 3.0.

RRID: SCR_016288

**Availability of supporting data and material**

The download URL of public datasets used in this study are available in Materials and Methods section.

The R code scripts used to generate testing data during this study are available in github, <https://github.com/Menggg/BLINK>

**Additional files**

Table S1. Properties of real genotypes and parameters of phenotype simulation.

Table S2. The information of operating system and machine configuration of computers for computing speed evaluation.

Table S3. The comparison of command lines between BLINK and PLINK.

**Supplemental Figure 1.** Population structure revealed by the first three principal components. The principal components (PC) were derived from all the available markers in each of the five species. Pair-wise relationship is displayed by the left column (PC1 vs. PC2), middle column (PC1 vs. PC3) and the right column (PC2 vs. PC3).

**Supplemental Figure 2.** The distribution of real phenotypes data in maize, Arabidopsis thaliana, mouse and pig.

**Supplemental Figure 3.** Proportion of case and control for lung cancer. The dataset contained a total of 8,807 samples, including 4,962 lung cancer cases and 3,845 controls.

**Supplemental Figure 4.** Association studies of flowering time in Arabidopsis thaliana. Four GWAS methods were used, GLM (performed by PLINK), BOLT-LMM, FarmCPU, and BLINK. Flowering time at 16°C was measured on 193 Arabidopsis thaliana individuals, genotyped with 216,131 SNPs. GLM included the first three PCs as covariates to control population structure. The names of flowering time candidate genes with significant SNPs nearby were labeled on the BLINK plot. The distances between significant SNPs and candidate genes were also labeled. All candidate genes’ information came from The Arabidopsis Information Resource (<http://www.arabidopsis.org/index.jsp>).

**Supplemental Figure 5.** Association studies of lung cancer in human. Four GWAS methods were used, Logistic Regression (performed by PLINK), FarmCPU, BOLT-LMM, and BLINK. The East Asian lung cancer population included 8,807 samples; each sample was genotyped with 629,968 SNPs (filtered by Minor Allele Frequency > 0.05, leaving 444,758 SNPs for the association study). The names of lung cancer candidate genes (Qing et al., Nature Genetics, 44, 1330-1335, 2012) with significant SNPs nearby were labeled on the BLINK plot. The distances between significant SNPs and candidate genes were also labeled.

**Supplemental Figure 6.** Association studies of weight growth intercept in mouse. Four GWAS methods were used, GLM (performed by PLINK), FarmCPU, BOLT-LMM, and BLINK. The population included 1,940 samples; each sample was genotyped with 12,226 SNPs (filtered by Minor Allele Frequency > 0.05, leaving 10,432 SNPs for the association study). GLM included the first three PCs as covariates to control population structure. The names of weight growth intercept candidate genes and QTL with significant SNPs nearby were labeled on the BLINK plot. The distances between significant SNPs and candidate genes/QTL were also labeled. All QTLs’ information came from Mouse Genome Informatics (URL: <http://www.informatics.jax.org/>).

**Supplemental Figure 7.** Association studies of last rib backfat thickness in pig. Four GWAS methods were used, GLM (performed by PLINK), FarmCPU, BOLT-LMM, and BLINK. The population included 820 samples; each sample was genotyped with 64,212 SNPs (filtered by Minor Allele Frequency > 0.05, leaving 40,748 SNPs for the association study). GLM included the first three PCs as covariates to control population structure. The names of backfat thickness candidate genes and QTL with significant SNPs nearby were labeled in the BLINK plot. The distances between significant SNPs and candidate genes/QTL were also labeled. All QTLs’ information came from the Pig Quantitative Trait Locus Database (PigQTLdb, URL: <http://www.animalgenome.org/cgi-bin/QTLdb/SS/index>).

**Supplemental Figure 8.** ROC plot of Figure 2 with 1 KB window size to count false and true positives. Number of false and true positives were counted based on 1 KB-sized bins.

**Supplemental Figure 9.** ROC plot of Figure 2 with 1 bp window size to count false and true positives. Number of false and true positives were counted based on 1 bp-sized bins.

**Supplemental Figure 10.** Statistical power and area under curve to detect un-clustered causal genes. Statistical power was defined as the proportion of simulated QTNs detected at cost defined by either False Positive Rate (FDR) or Type I error. The two types of ROC curves are displayed separately for FDR (a) and Type I error (b). The AUC is also displayed separately for FDR (c) and versus Type I error (d). Four GWAS methods (BLINK, FarmCPU, BOLT-LMM and PLINK) were compared with phenotypes simulated from real genotypes in five species (human, maize, Arabidopsis thaliana, mouse, and pig). The simulated phenotypes had a heritability of 75%, controlled by 500 QTNs for human, 100 QTNs for maize and mouse, and 50 QTNs for Arabidopsis thaliana and pig. These QTNs were randomly sampled from the available SNPs without restriction. The number of false and true positives were counted based on 10KB-sized bins.

**Supplemental Figure 11.** ROC plot of Figure S4 with 1 KB window size to count false and true positives. Number of false and true positives were counted based on 1 KB-sized bins.

**Supplemental Figure 12.** ROC plot of Figure S4 with 1 bp window size to count false and true positives. Number of false and true positives were counted based on 1 bp-sized bins.

**Supplemental Figure 13.** The performance of three model selection criteria. The three model selection criteria are Bayesian Information Criterion (BIC), Akaike Information Criterion (AIC), and Extended Bayesian Information Criterion (EBIC). The performance was evaluated as statistical power vs. False Discovery Rate (FDR). Statistical power was defined as the proportion of simulated Quantitative Trait Nucleotides (QTNs) detected at different levels of FDR. The simulated QTNs were sampled from the real genotypes in five species (human, maize, Arabidopsis thaliana, mouse, and pig). The simulated phenotypes had a heritability of 75%, controlled by 500 QTNs for human, 100 QTNs for maize and mouse, and 50 QTNs for Arabidopsis thaliana and pig. These QTNs were randomly sampled from the available Single Nucleotide Polymorphism (SNPs) with the restriction that every two QTNs were clustered within 300 Kb distance. BIC overperformed other two model selection criteria.

**Supplemental Figure 14.** Impact of cutoff to exclude correlated markers on statistical power. The impact was evaluated as statistical power at different levels of False Discovery Rate (FDR). Statistical power was defined as the proportion of simulated Quantitative Trait Nucleotides (QTNs) detected at different levels of FDR. The simulated QTNs were sampled from the real genotypes in five species (human, maize, Arabidopsis thaliana, mouse, and pig). The simulated phenotypes had a heritability of 75%, controlled by 500 QTNs for human, 100 QTNs for maize and mouse, and 50 QTNs for Arabidopsis thaliana and pig. These QTNs were randomly sampled from the available Single Nucleotide Polymorphism (SNPs) with the restriction that every two QTNs were clustered within 300 Kb distance. The cutoff was varied from 0.1 to 0.9 for excluding genetic markers sorted on the strength of association with phenotypes. Higher cutoff leads to more markers as covariates in the model for next iteration of association tests.

**Supplemental Figure 15.** Identical P values by using BLINK C version and R version. The P values were the association tests on real phenotypes in four species. The phenotypes are (a) last rib backfat thickness (pig), (b) lung cancer (human), (c) weight growth intercept (mouse), and (d) flowering time (Arabidopsis). The P values are displayed as –log10(P value).

**Supplemental Figure 16.** Snapshot of random selected Manhattan plots out of 100 replicates. The Manhattan plots were based on the P values by using BLINK on phenotypes simulated from real genotypes in five species (human, maize, Arabidopsis thaliana, mouse, and pig). The simulated phenotypes had a heritability of 75%, controlled by 500 QTNs for human, 100 QTNs for maize and mouse, and 50 QTNs for Arabidopsis thaliana and pig. These QTNs with gray dots and circles were randomly sampled from the available Single Nucleotide Polymorphism (SNPs) with the restriction that every two QTNs were clustered within 100 Kb distance. The green lines indicated the Bonferroni multiple test threshold.

**Supplemental Figure 17.** Effects of number of principal components (PCs) and kinship pruning. Fitting two PCs and their products had much worse control of P value inflation due to population stratification compared with fitting ten PCs for association study on maize flowering time. The inflation was further improved by kinship pruning in PLINK at cutoff of 0.5, which reduced number of samples from 2,279 to 1,218. The number of significant SNPs (Bonferroni cutoff of α = 0.01) were reduced from 48,194 SNPs with two PCs and their product, to 2,671 SNPs with ten PCs, and to 211 SNPs with ten PCs plus kinship pruning.

**Declarations**

**Abbreviations**

GWAS: genome-wide association studies;

FarmCPU: fixed and random model firculating probability unification;

QTNs: quantitative trait nucleotides;

FEM: fixed effect model;

REM: random effect model;

GLM: general linear model;

PCA: principal component analysis;

LD: linkage disequilibrium;

MLM: mixed minear model;

EMMA: efficient mixed model association**;**

ML: maximum likelihood;

REML: restricted maximum likelihood;

EM: expectation and maximization;

P3D: population parameters previously petermined;

EMMAX: EMMA eXpedited;

GEMMA: genome-wide efficient mixed-model association;

CMLM: compressed MLM;

FaST-LMM: factored spectrally transformed linear mixed models;

SUPER: settlement of MLM under progressively exclusive relationship;

MLMM: multi-locus mixed-model;

BIC:bayesian information criteria;

FDR: false discovery rate;

ROC: receiver operating characteristic;

AUC: area under curve;

AIC: akaike information criterion;

EBIC: extended BIC;

FOAM: F-one association mapping;

CPU: central processing unit;

BLINK: bayesian-information and linkage-disequilibrium iteratively nested keyway;

SNP: single nucleotide polymorphism;

**Competing interests**

The authors declare that they do not have competing financial interests.

**Funding**

This material is based upon work that is supported by an Emerging Research Issues Internal Competitive Grant from the Agricultural Research Center in the College of Agricultural, Human, and Natural Resource Sciences at Washington State University; the Washington Grain Commission (Endowment and award number 126593); the National Science Foundation (Award number DBI 1661348), the National Institute of Food and Agriculture, U.S. Department of Agriculture (awards 2015-05798 and 2016-68004-24770).

**Author contributions**

ZZ conceptualized the study and wrote the manuscript. The concepts were implemented by MH in C language (BLINK-C), and by YZ in R language (BLINK-R). MH, XL, YZ, and RMS performed the data analyses.

**Acknowledgements**

The authors thank Dr. Linda R. Klein for valuable writing advice and editing the manuscript.

**Ethics statement**

Any opinions, findings, conclusions, or recommendations expressed in this publication are those of the authors and do not necessarily reflect the views of the funding agencies. All datasets analyzed herein were published previously. This study did not involve samples from humans or animals.

**References**

1. Georges M. Towards sequence-based genomic selection of cattle. Nat Genet [Internet]. 2014;46:807–9.

2. Shendure J, Mitra RD, Varma C, Church GM. Advanced sequencing technologies: methods and goals. Nat Rev Genet. 2004;5:335–44.

3. Frazer KA, Murray SS, Schork NJ, Topol EJ. Human genetic variation and its contribution to complex traits. Nat Rev Genet. 2009;10(4):241–51.

4. Glazier AM, Nadeau JH, Aitman TJ. Finding genes that underlie complex traits. Science. 2002;298:2345–9.

5. Lan Q, Hsiung C a, Matsuo K, Hong Y-C, Seow A, Wang Z, et al. Genome-wide association analysis identifies new lung cancer susceptibility loci in never-smoking women in Asia. Nat Genet. 2012;44:1330–5.

6. Moonesinghe R, Khoury MJ, Janssens ACJW. Most Published Research Findings Are False—But a Little Replication Goes a Long Way. PLoS Med. 2007;4:4.

7. Larsson SJ, Lipka AE, Buckler ES. Lessons from Dwarf8 on the Strengths and Weaknesses of Structured Association Mapping. PLoS Genet. 2013;9.

8. Ioannidis JPA. Why most published research findings are false. PLoS Med. 2005;2:0696–701.

9. Yu J, Pressoir G, Briggs WH, Vroh Bi I, Yamasaki M, Doebley JF, et al. A unified mixed-model method for association mapping that accounts for multiple levels of relatedness. Nat Genet. 2006;38:203–8.

10. Atwell S, Huang YS, Vilhjálmsson BJ, Willems G, Horton M, Li Y, et al. Genome-wide association study of 107 phenotypes in Arabidopsis thaliana inbred lines. Nature. 2010;465:627–31.

11. Yang J, Zaitlen N a, Goddard ME, Visscher PM, Price AL. Advantages and pitfalls in the application of mixed-model association methods. Nat Genet. Nature Publishing Group; 2014;46:100–6.

12. Pritchard JK, Stephens M, Donnelly P. Inference of population structure using multilocus genotype data. Genetics. 2000;155:945–59.

13. Pritchard JK, Stephens M, Rosenberg NA, Donnelly P. Association mapping in structured populations. Am J Hum Genet. 2000;67:170–81.

14. Price AL, Patterson NJ, Plenge RM, Weinblatt ME, Shadick NA, Reich D. Principal components analysis corrects for stratification in genome-wide association studies. Nat Genet. 2006;38:904–9.

15. Price AL, Zaitlen NA, Reich D, Patterson N. New approaches to population stratification in genome-wide association studies. Nat Rev Genet. 2010;11:459–63.

16. de los Campos G, Gianola D, Allison DB. Predicting genetic predisposition in humans: the promise of whole-genome markers. Nat Rev Genet. 2010/11/04. 2010;11:880–6.

17. Meuwissen TH, Hayes BJ, Goddard ME. Prediction of total genetic value using genome-wide dense marker maps. Genetics. 2001;157:1819–29.

18. Loh P, Tucker G, Bulik-sullivan BK, Vilhj BJ. Efficient Bayesian mixed model analysis increases association power in large cohorts. Nat Genet. 2014;47:284–90.

19. Kang HM, Zaitlen NA, Wade CM, Kirby A, Heckerman D, Daly MJ, et al. Efficient control of population structure in model organism association mapping. Genetics. 2008;178:1709–23.

20. Kang HM, Sul JH, Service SK, Zaitlen NA, Kong S-Y, Freimer NB, et al. Variance component model to account for sample structure in genome-wide association studies. Nat Genet. 2010;42:348–54.

21. Zhang Z, Ersoz E, Lai CQ, Todhunter RJ, Tiwari HK, Gore M a, et al. Mixed linear model approach adapted for genome-wide association studies. Nat Genet. Nature Publishing Group; 2010;42:355–60.

22. Zhou X, Stephens M. Genome-wide efficient mixed-model analysis for association studies. Nat. Genet. 2012;44:821–4.

23. Lippert C, Listgarten J, Liu Y, Kadie CM, Davidson RI, Heckerman D. FaST linear mixed models for genome-wide association studies. Nat. Methods. 2011;8:833–5.

24. Svishcheva GR, Axenovich TI, Belonogova NM, van Duijn CM, Aulchenko YS. Rapid variance components–based method for whole-genome association analysis. Nat. Genet. 2012;44:1166–70.

25. Li M, Liu X, Bradbury P, Yu J, Zhang Y-M, Todhunter RJ, et al. Enrichment of statistical power for genome-wide association studies. BMC Biol. 2014;12:73.

26. Listgarten J, Lippert C, Kadie CM, Davidson RI, Eskin E, Heckerman D. Improved linear mixed models for genome-wide association studies. Nat. Methods. 2012;9:525–6.

27. Wang Q, Tian F, Pan Y, Buckler ES, Zhang Z. A super powerful method for genome wide association study. PLoS One. 2014;9:e107684.

28. Segura V, Vilhjálmsson BJ, Platt A, Korte A, Seren Ü, Long Q, et al. An efficient multi-locus mixed-model approach for genome-wide association studies in structured populations. Nat. Genet. 2012;44:825–30.

29. Liu X, Huang M, Fan B, Buckler ES, Zhang Z. Iterative Usage of Fixed and Random Effect Models for Powerful and Efficient Genome-Wide Association Studies. PLoS Genet. Public Library of Science; 2016;12:e1005767.

30. Purcell S, Neale B, Todd-Brown K, Thomas L, Ferreira MAR, Bender D, et al. PLINK: A Tool Set for Whole-Genome Association and Population-Based Linkage Analyses. Am J Hum Genet. 2007;81:559–575.

31. Loh P, Tucker G, Bulik-sullivan BK, Vilhj BJ. Efficient Bayesian mixed model analysis increases association power in large cohorts. Nat. Genet. 2014;47:284–90.

32. Akaike H. A New Look at the Statistical Model Identification. IEEE Trans Automat Contr. 1974;19:716–23.

33. Chen J, Chen Z. Extended Bayesian information criteria for model selection with large model spaces. Biometrika. 2008;95:759–71.

34. Buckler ES, Holland JB, Bradbury PJ, Acharya CB, Brown PJ, Browne C, et al. The genetic architecture of maize flowering time. Science. 2009;325:714–8.

35. Romero Navarro JA, Willcox M, Burgueño J, Romay C, Swarts K, Trachsel S, et al. A study of allelic diversity underlying flowering-time adaptation in maize landraces. Nat Genet. 2017;49:476-80.

36. Chang CC, Chow CC, Tellier LCAM, Vattikuti S, Purcell SM, Lee JJ. Second-generation PLINK: Rising to the challenge of larger and richer datasets. Gigascience. 2015;4:7.

37. Romay MC, Millard MJ, Glaubitz JC, Peiffer JA, Swarts KL, Casstevens TM, et al. Comprehensive genotyping of the USA national maize inbred seed bank. Genome Biol. 2013;14:R55.

38. Neves HHR, Carvalheiro R, Queiroz SA. A comparison of statistical methods for genomic selection in a mice population. BMC Genet. 2012;13:100.

39. Fan B, Onteru SK, Du ZQ, Garrick DJ, Stalder KJ, Rothschild MF. Genome-wide association study identifies loci for body composition and structural soundness traits in pigs. PLoS One. 2011;6:e0014726.

40. Tang Y, Liu X, Wang J, Li M, Wang Q, Tian F, et al. GAPIT Version 2: An Enhanced Integrated Tool for Genomic Association and Prediction. Plant Genome. 2016;9.

41. Lipka AE, Tian F, Wang Q, Peiffer J, Li M, Bradbury PJ, et al. GAPIT: Genome association and prediction integrated tool. Bioinformatics. 2012;28:2397-9.

**Figures**

**Figure 1.** Limitation of the bin approach and proposed solution. Quantitative Trait Nucleotides (QTNs) are rarely distributed evenly throughout the genome, as required by the bin approach used in FarmCPU. The most significant marker from each bin, indicated by the filled red and black circles in (**a**) and (**b**), is selected as a pseudo QTN if it passes a threshold (dash lines across vertical axes). A pseudo QTN could be false (filled black circle) if the bins (separated by the vertical lines) are too small **(a)**, or true, but not selected (open circle) if the bins are too big (**b**), as illustrated by comparing the true QTNs (red triangles) positioned along the horizontal axis in (**c**). Our alternative method is to sort the M SNPs first and filter them out if their P values are larger than a threshold (α). Among the m SNPs kept, additional SNPs are removed if their correlation (r) with the first SNP (S_1_^*^) is larger than a threshold (β). This process is repeated to select S_2_^*^, S_3_^*^,…, until the last SNP S_t_^*^ is selected (**d**). As the t remaining SNPs are sorted, we fit the first k of them in a Fixed Effect Model (FEM) **(e)** and examine the corresponding twice negative log likelihood (-2LL) and Bayesian Information Criteria (BIC) **(f)**. As more SNPs are fitted, -2LL continually improves (blue line), while BIC reverses (red line) because BIC applies a penalty with increasing numbers of SNPs. The set of k SNPs that give the best BIC are used as pseudo QTNs and fitted as covariates in another FEM to test all SNPs, one (s_i_) at a time, as described by the conceptual model (**g**). This process (**d** to **g**) is iterated until the pseudo QTNs remain the same. We named this alternative solution the Bayesian-information and Linkage-disequilibrium Iteratively Nested Keyway (BLINK) method**.**

**Figure 2.** Statistical power and area under curve to detect clustered causal genes. Statistical power was defined as the proportion of simulated Quantitative Trait Nucleotides (QTNs) detected at cost, defined by either False Discovery Rate (FDR) or Type I error. The two types of Receiver Operating Characteristic (ROC) curves are displayed separately for FDR **(a)** and Type I error **(b)**. The Area Under the Curves (AUC) are also displayed separately for FDR **(c)** and Type I error **(d)**. Four GWAS methods (BLINK, FarmCPU, PLINK, and BOLT-LMM) were compared with phenotypes simulated from real genotypes in five species (human, maize, *Arabidopsis thaliana*, mouse, and pig). The simulated phenotypes had a heritability of 75%, controlled by 500 QTNs for human, 100 QTNs for maize and mouse, and 50 QTNs for *Arabidopsis thaliana* and pig. These QTNs were randomly sampled from the available Single Nucleotide Polymorphism (SNPs), with the restriction that every two QTNs were clustered within a distance of 300 Kb.

**Figure 3.** GWAS of flowering time (days to silk) in maize. The performance of four GWAS methods, BLINK, FarmCPU, BOLT-LMM, and PLINK, are compared. The population included 2,648 individuals genotyped with 397,323 SNPs, after filtering out SNPs with a Minor Allele Frequency of 5% or less. All methods included the first two PCs and their products as covariates. The names of flowering-time candidate genes and NAM QTL that are surrounded by significant SNPs are labeled on the top, including the distances between significant SNPs and candidate genes/NAM QTL.

**Figure 4.** Enrichment of associated SNPs identified by BLINK and FarmCPU. SNPs associated with maize flowering time were identified by BLINK and FarmCPU, using the Ames population containing 2,279 lines. These SNPs were classified as the FarmCPU unique SNPs (5), common SNPs (9) and BLINK unique SNPs (40). The enrichment was performed on the SNPs that overlapped (within 50,000 base pairs) with the 1,003 flowering candidate genes identified by a separate population containing 4,471 landraces (**a**). The null probability distributions are illustrated as the histograms of randomly sampled sets of 5, 9, and 40 overlapping SNPs from the maize genome (**b**). The FarmCPU unique SNPs were not enriched. The common SNPs and BLINK unique SNPs were significantly enriched. The null probability was less than 1% for randomly sampling five SNPs with four or more overlapped with the 1003 candidate genes. Similarly, the null probability was less than 3% for randomly sampling forty SNPs with eight or more overlapped with the 1003 candidate genes.

**Figure 5.** BLINK performances on computing time and parallelization. The computing times using BLINK-C and BLINK-R are compared with PLINK (version 1.90) and FarmCPU (**a**) on synthetic datasets with duplication on the original dataset containing 8,800 individuals genotyped with one-half million markers. BLINK-C can conduct parallel computation by using multiple central processing unit (CPU) cores. Different computers under different platforms were used to evaluate the parallelization efficiency of BLINK-C (**b**). The efficiency is illustrated as the ratio of computing time of a single core to the computing time of multiple cores.
