# Supplementary material for: BLINK: a package for the next level of genome-wide association studies with both individuals and markers in the millions
Source: Gigascience. 2018 Dec 11;8(2):giy154. doi: 10.1093/gigascience/giy154 (PMC6365300; doi:10.1093/gigascience/giy154)
Supplement: Supplemental Files [file giy154_supplemental_files.zip › S10_Figure.docx]

**
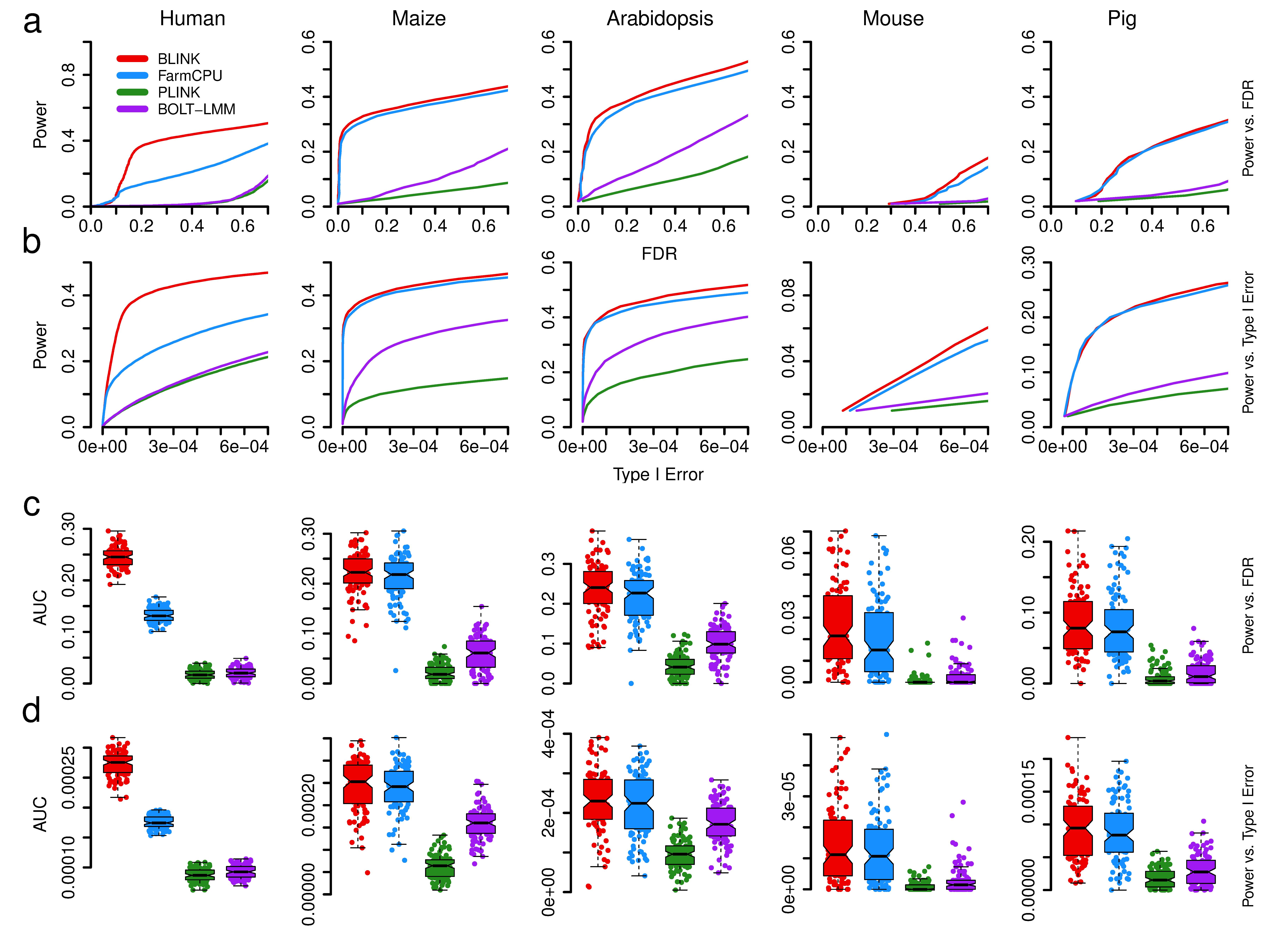
**

**S10 Figure**. **Statistical power and area under curve to detect un-clustered causal genes**. Statistical power was defined as the proportion of simulated QTNs detected at cost defined by either False Positive Rate (**FDR**) or Type I error. The two types of ROC curves are displayed separately for FDR **(a)** and Type I error **(b)**. The AUC is also displayed separately for FDR **(c)** and versus Type I error **(d)**. Four GWAS methods (BLINK, FarmCPU, BOLT-LMM and PLINK) were compared with phenotypes simulated from real genotypes in five species (human, maize, *Arabidopsis thaliana*, mouse, and pig). The simulated phenotypes had a heritability of 75%, controlled by 500 QTNs for human, 100 QTNs for maize and mouse, and 50 QTNs for *Arabidopsis thaliana* and pig. These QTNs were randomly sampled from the available SNPs without restriction. The number of false and true positives were counted based on 10KB-sized bins.
