# Supplementary material for: BLINK: a package for the next level of genome-wide association studies with both individuals and markers in the millions
Source: Gigascience. 2018 Dec 11;8(2):giy154. doi: 10.1093/gigascience/giy154 (PMC6365300; doi:10.1093/gigascience/giy154)
Supplement: Supplemental Files [file giy154_supplemental_files.zip › S11_Figure.docx]

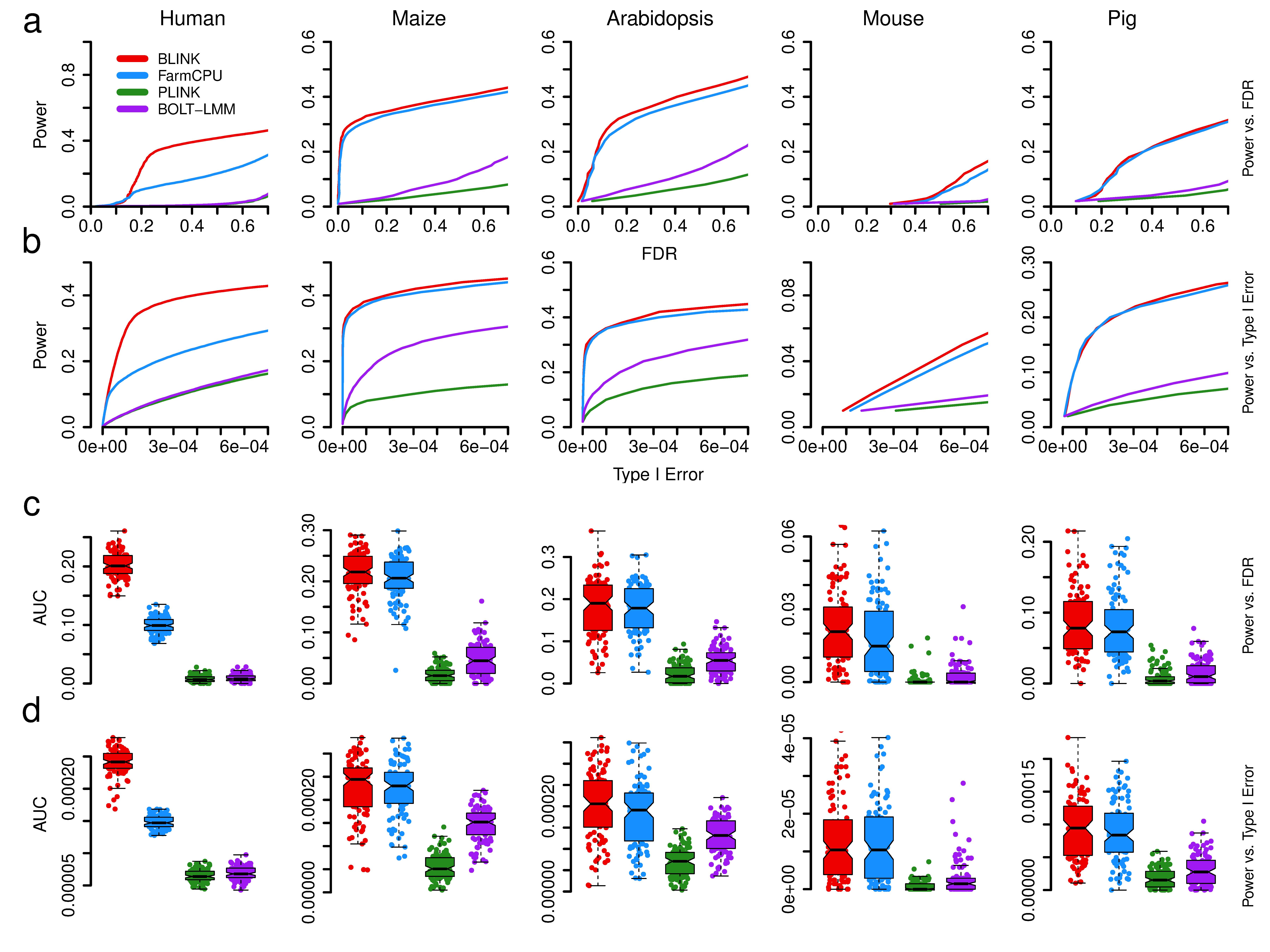


**S11 Figure**. **ROC plot of Figure S4 with 1 KB window size to count false and true positives.** Number of false and true positives were counted based on 1 KB-sized bins.
