# Supplementary material for: BLINK: a package for the next level of genome-wide association studies with both individuals and markers in the millions
Source: Gigascience. 2018 Dec 11;8(2):giy154. doi: 10.1093/gigascience/giy154 (PMC6365300; doi:10.1093/gigascience/giy154)
Supplement: Supplemental Files [file giy154_supplemental_files.zip › S13_Figure.docx]

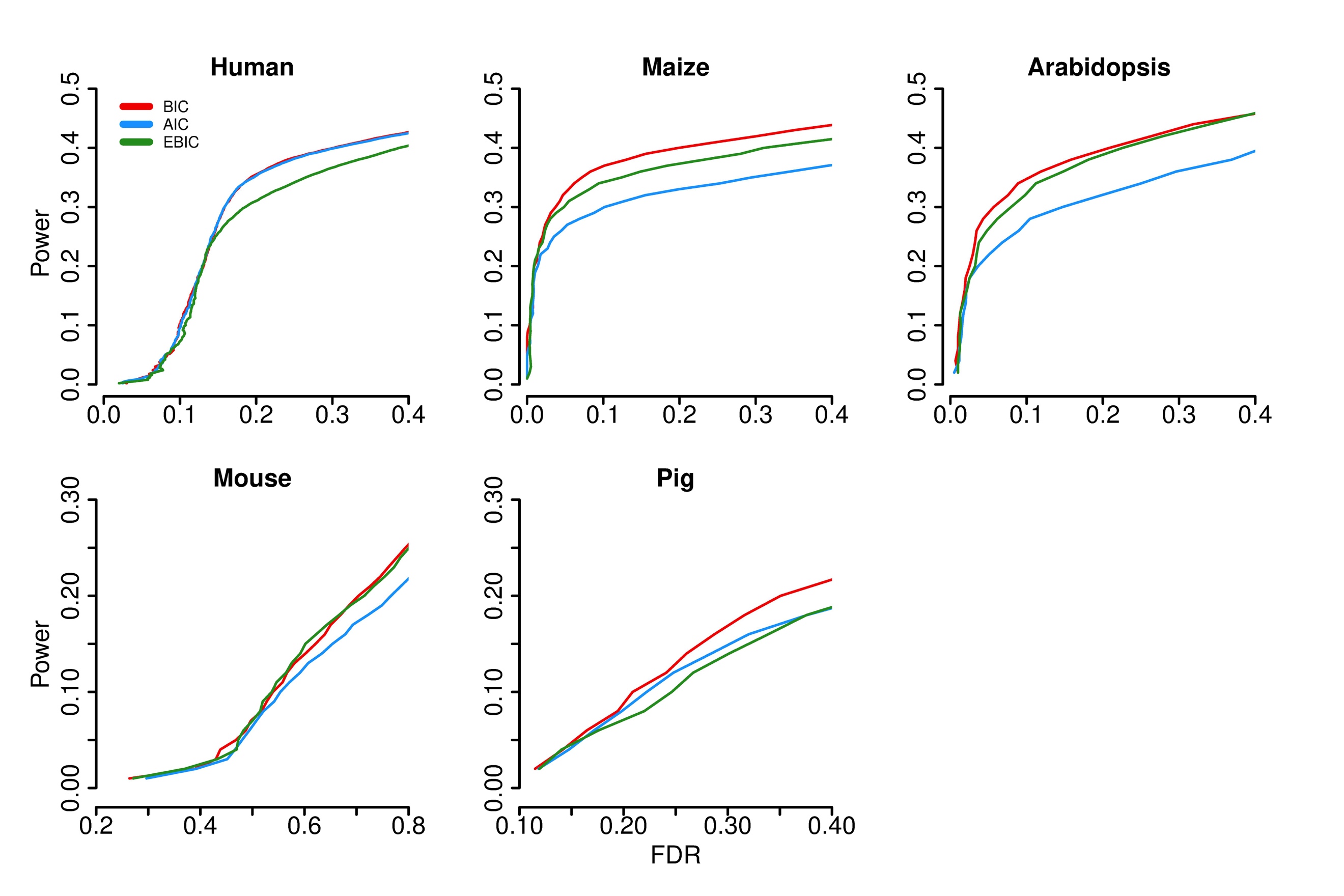


**S13 Figure. The performance of three model selection criteria.** The three model selection criteria are Bayesian Information Criterion (BIC), Akaike Information Criterion (AIC), and Extended Bayesian Information Criterion (EBIC). The performance was evaluated as statistical power vs. False Discovery Rate (FDR). Statistical power was defined as the proportion of simulated Quantitative Trait Nucleotides (QTNs) detected at different levels of FDR. The simulated QTNs were sampled from the real genotypes in five species (human, maize, *Arabidopsis thaliana*, mouse, and pig). The simulated phenotypes had a heritability of 75%, controlled by 500 QTNs for human, 100 QTNs for maize and mouse, and 50 QTNs for *Arabidopsis thaliana* and pig. These QTNs were randomly sampled from the available Single Nucleotide Polymorphism (SNPs) with the restriction that every two QTNs were clustered within 300 Kb distance. BIC overperformed other two model selection criteria.
