# Supplementary material for: BLINK: a package for the next level of genome-wide association studies with both individuals and markers in the millions
Source: Gigascience. 2018 Dec 11;8(2):giy154. doi: 10.1093/gigascience/giy154 (PMC6365300; doi:10.1093/gigascience/giy154)
Supplement: Supplemental Files [file giy154_supplemental_files.zip › S15_Figure.docx]

**
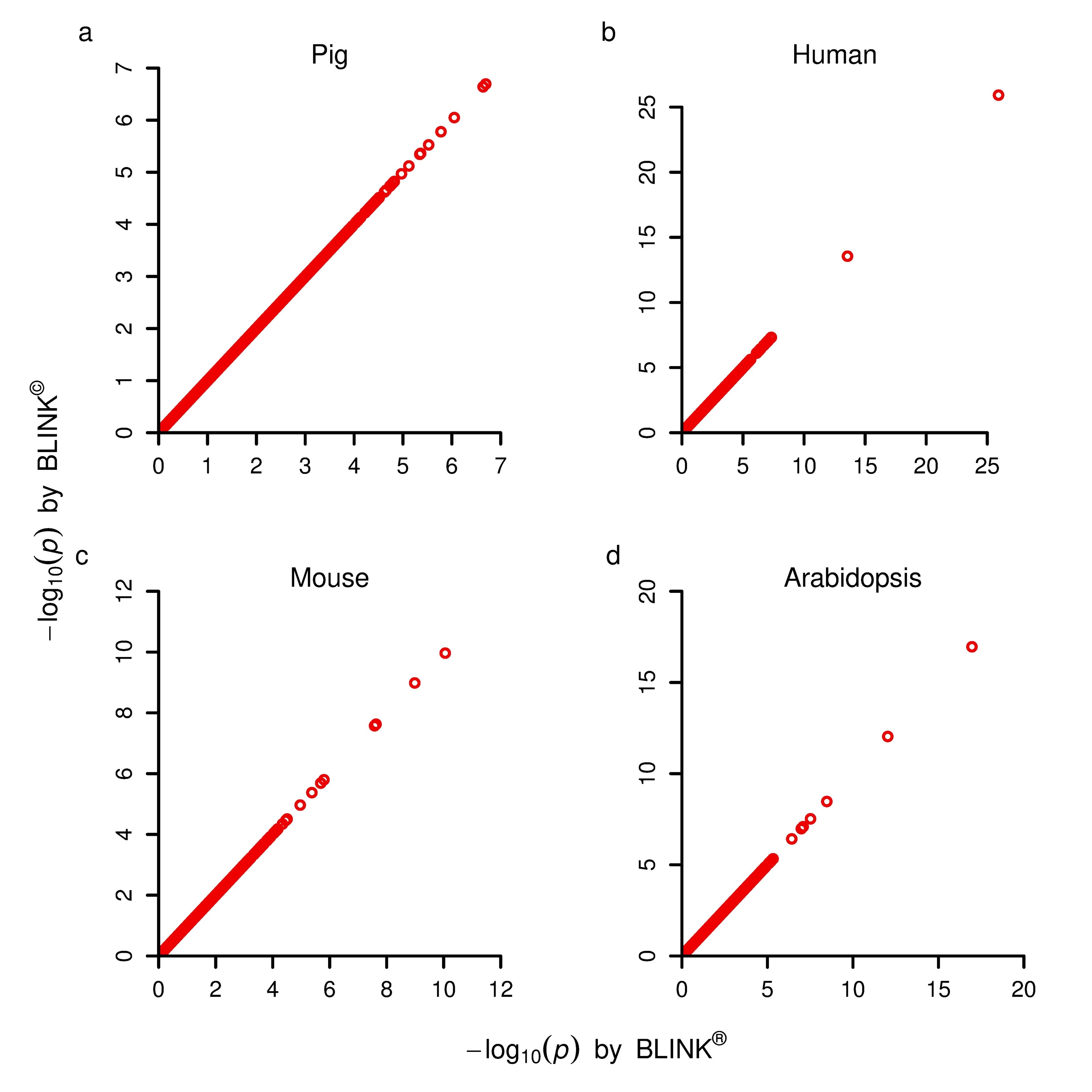
**

**S15 Figure. Identical P values by using BLINK C version and R version.** The P values were the association tests on real phenotypes in four species. The phenotypes are (a) last rib backfat thickness (pig), (b) lung cancer (human), (c) weight growth intercept (mouse), and (d) flowering time (Arabidopsis). The P values are displayed as –log_10_(*P* value).
