# Supplementary material for: BLINK: a package for the next level of genome-wide association studies with both individuals and markers in the millions
Source: Gigascience. 2018 Dec 11;8(2):giy154. doi: 10.1093/gigascience/giy154 (PMC6365300; doi:10.1093/gigascience/giy154)
Supplement: Supplemental Files [file giy154_supplemental_files.zip › S16_Figure.docx]

**
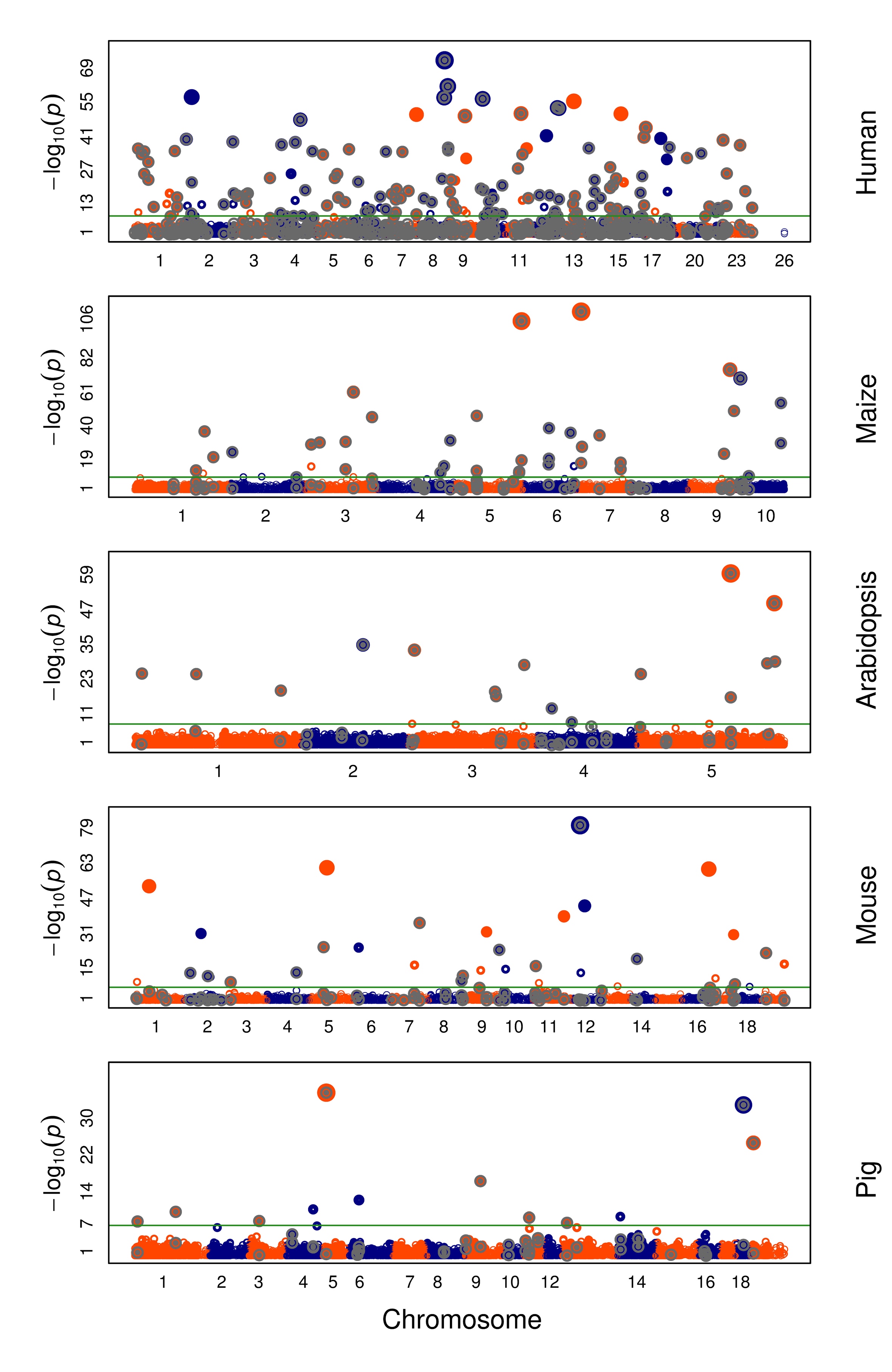
**

**S16 Figure. Snapshot of random selected Manhattan plots out of 100 replicates.** The Manhattan plots were based on the P values by using BLINK on phenotypes simulated from real genotypes in five species (human, maize, *Arabidopsis thaliana*, mouse, and pig). The simulated phenotypes had a heritability of 75%, controlled by 500 QTNs for human, 100 QTNs for maize and mouse, and 50 QTNs for *Arabidopsis thaliana* and pig. These QTNs with gray dots and circles were randomly sampled from the available Single Nucleotide Polymorphism (SNPs) with the restriction that every two QTNs were clustered within 100 Kb distance. The green lines indicated the Bonferroni multiple test threshold.
