# Supplementary material for: BLINK: a package for the next level of genome-wide association studies with both individuals and markers in the millions
Source: Gigascience. 2018 Dec 11;8(2):giy154. doi: 10.1093/gigascience/giy154 (PMC6365300; doi:10.1093/gigascience/giy154)
Supplement: Supplemental Files [file giy154_supplemental_files.zip › S17_Figure.docx]

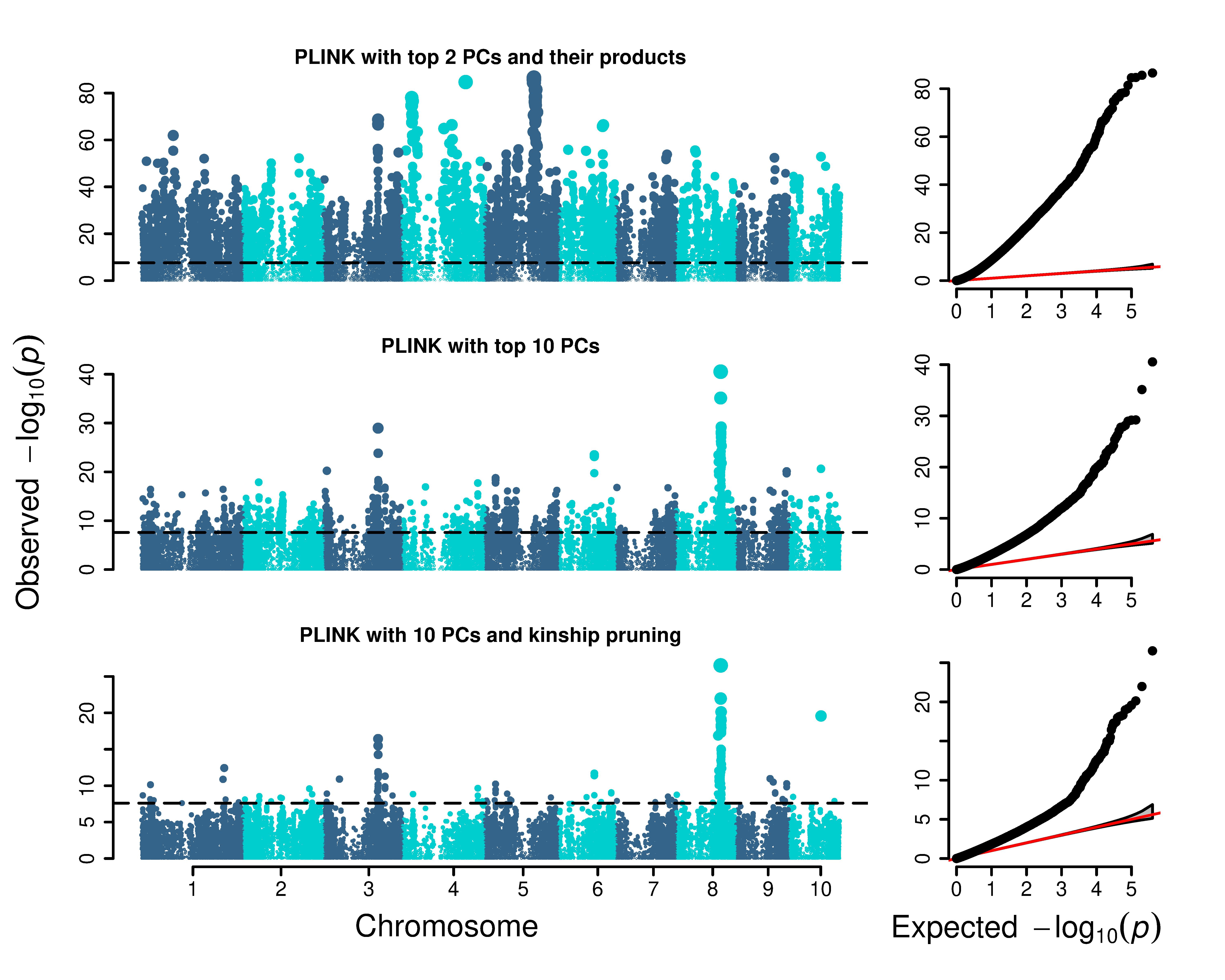


**S17 Figure. Effects of number of principal components (PCs) and kinship pruning.** Fitting two PCs and their products had much worse control of P value inflation due to population stratification compared with fitting ten PCs for association study on maize flowering time. The inflation was further improved by kinship pruning in PLINK at cutoff of 0.5, which reduced number of samples from 2,279 to 1,218. The number of significant SNPs (Bonferroni cutoff of α = 0.01) were reduced from 48,194 SNPs with two PCs and their product, to 2,671 SNPs with ten PCs, and to 211 SNPs with ten PCs plus kinship pruning.
