# Supplementary material for: BLINK: a package for the next level of genome-wide association studies with both individuals and markers in the millions
Source: Gigascience. 2018 Dec 11;8(2):giy154. doi: 10.1093/gigascience/giy154 (PMC6365300; doi:10.1093/gigascience/giy154)
Supplement: Supplemental Files [file giy154_supplemental_files.zip › S1_Figure.docx]

**
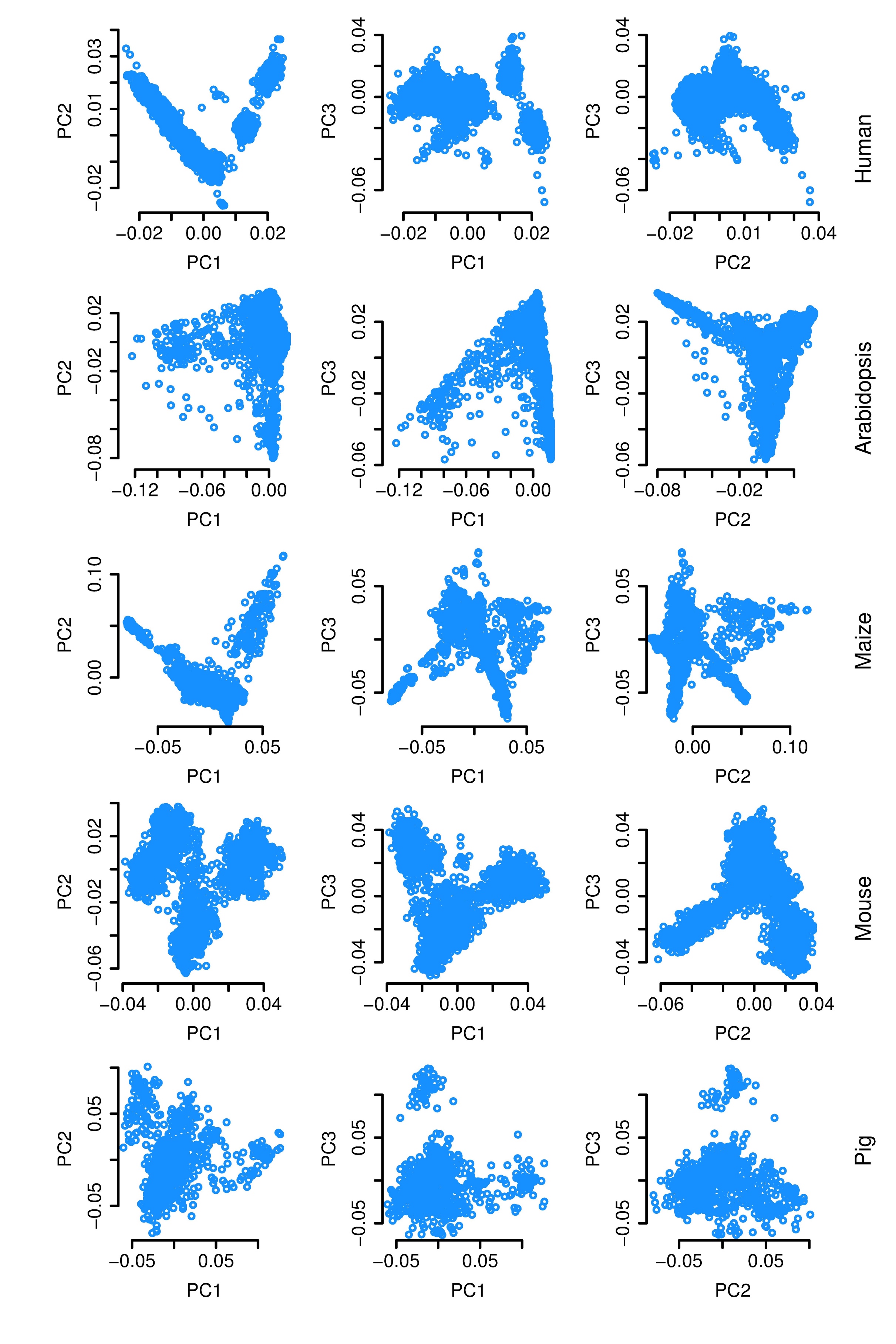
**

**S1 Figure. Population structure revealed by the first three principal components.** The principal components (PC) were derived from all the available markers in each of the five species. Pair-wise relationship is displayed by the left column (PC1 vs. PC2), middle column (PC1 vs. PC3) and the right column (PC2 vs. PC3).
