# Supplementary material for: BLINK: a package for the next level of genome-wide association studies with both individuals and markers in the millions
Source: Gigascience. 2018 Dec 11;8(2):giy154. doi: 10.1093/gigascience/giy154 (PMC6365300; doi:10.1093/gigascience/giy154)
Supplement: Supplemental Files [file giy154_supplemental_files.zip › S3_Figure.docx]

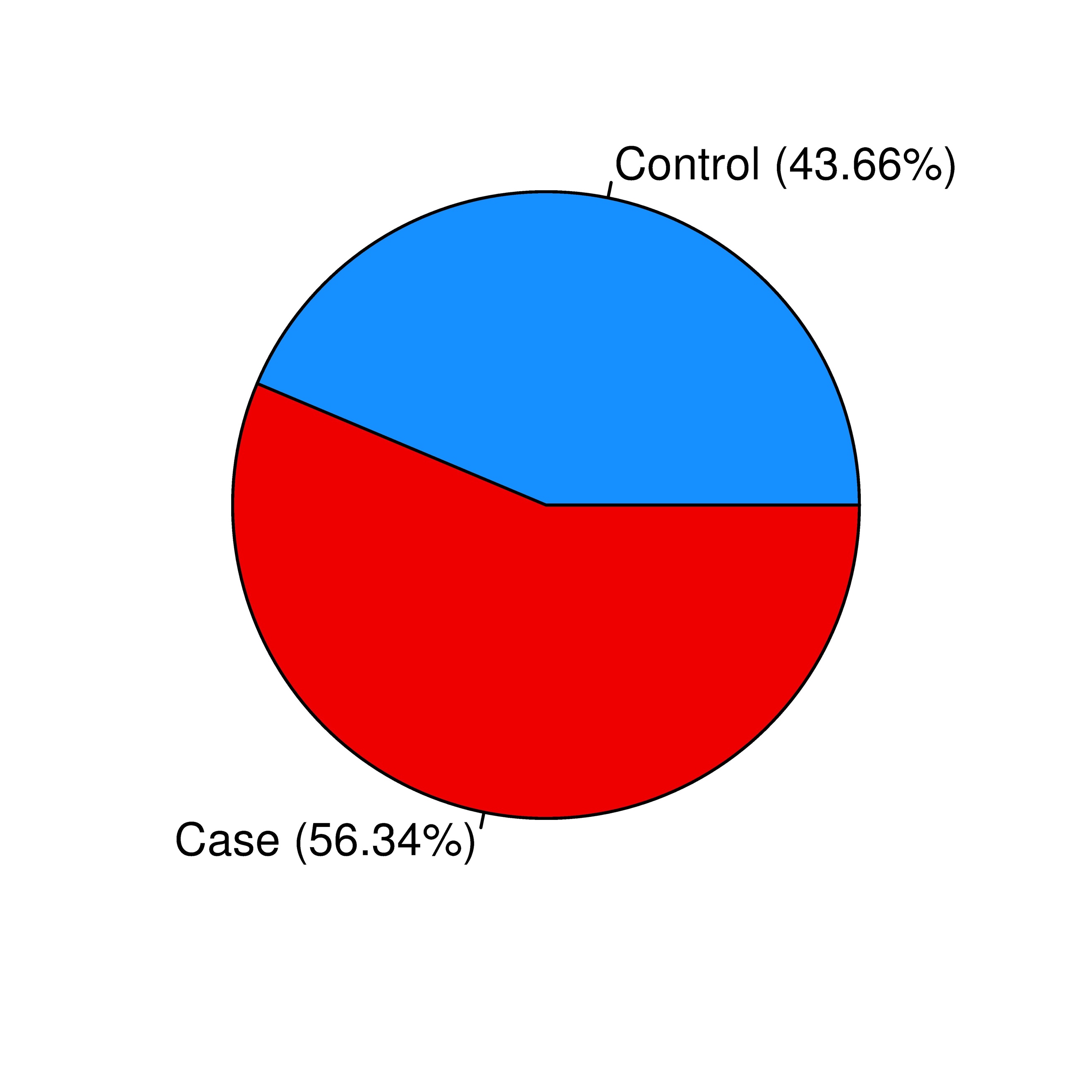


**S3 Fig.** Proportion of case and control for lung cancer. The dataset contained a total of 8,807 samples, including 4,962 lung cancer cases and 3,845 controls.
