# Supplementary material for: BLINK: a package for the next level of genome-wide association studies with both individuals and markers in the millions
Source: Gigascience. 2018 Dec 11;8(2):giy154. doi: 10.1093/gigascience/giy154 (PMC6365300; doi:10.1093/gigascience/giy154)
Supplement: Supplemental Files [file giy154_supplemental_files.zip › S4_Figure.docx]

**
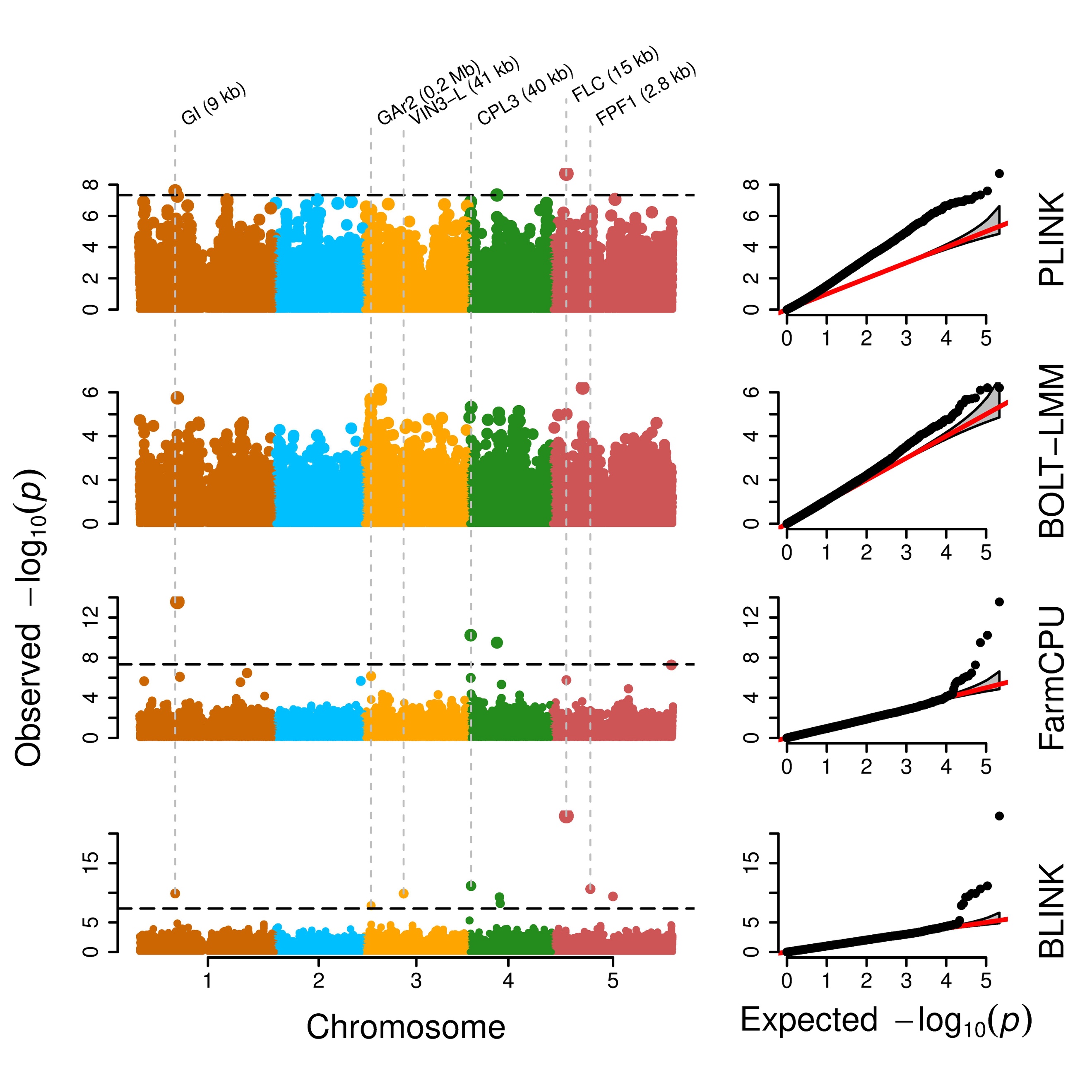
**

**S4 Fig. Association studies of flowering time in *Arabidopsis thaliana*.** Four GWAS methods were used, GLM (performed by PLINK), BOLT-LMM, FarmCPU, and BLINK. Flowering time at 16°C was measured on 193 *Arabidopsis thaliana* individuals, genotyped with 216,131 SNPs. GLM included the first three PCs as covariates to control population structure. The names of flowering time candidate genes with significant SNPs nearby were labeled on the BLINK plot. The distances between significant SNPs and candidate genes were also labeled. All candidate genes’ information came from The Arabidopsis Information Resource (<http://www.arabidopsis.org/index.jsp>).
