# Supplementary material for: BLINK: a package for the next level of genome-wide association studies with both individuals and markers in the millions
Source: Gigascience. 2018 Dec 11;8(2):giy154. doi: 10.1093/gigascience/giy154 (PMC6365300; doi:10.1093/gigascience/giy154)
Supplement: Supplemental Files [file giy154_supplemental_files.zip › S5_Figure.docx]

**
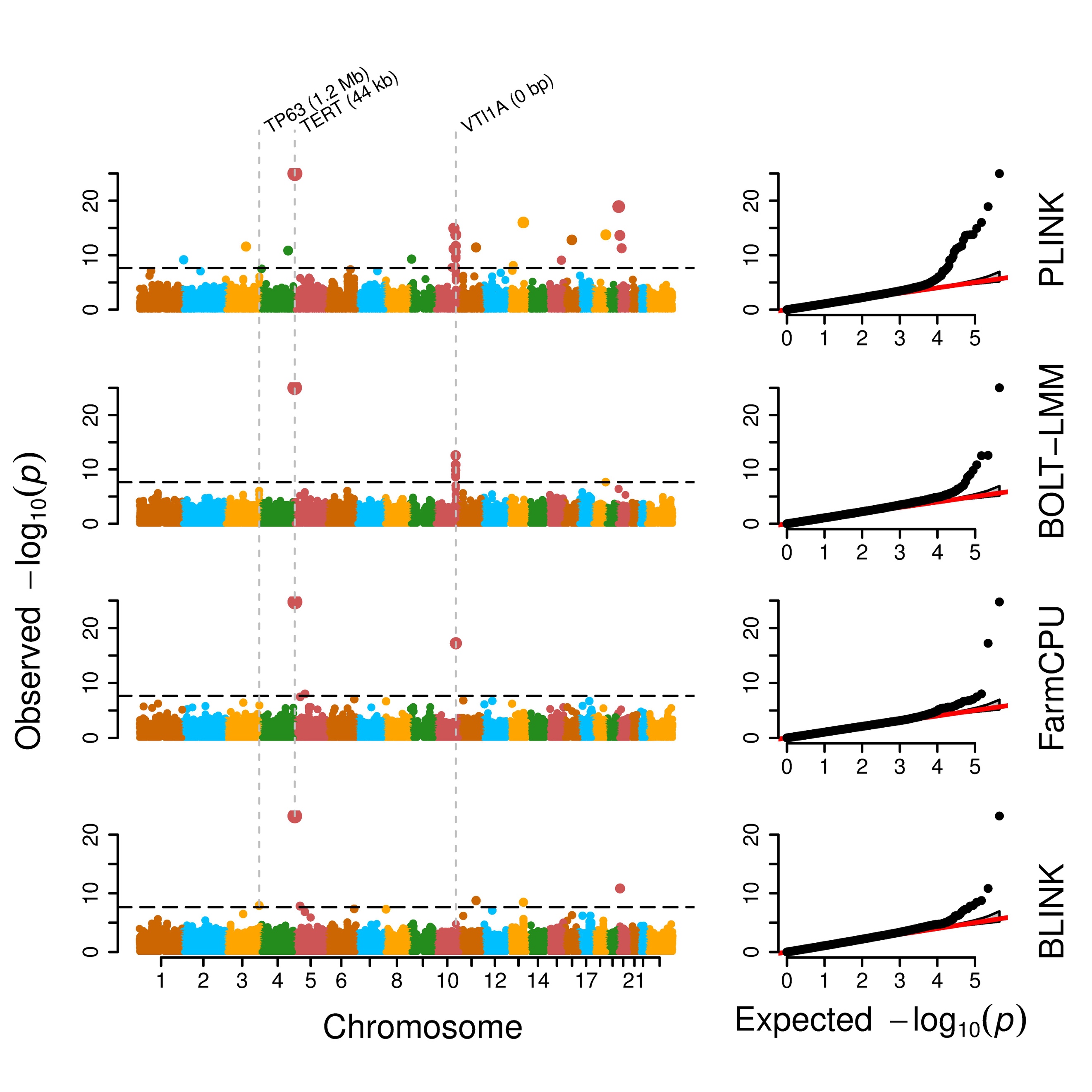
**

**S5 Fig. Association studies of lung cancer in human.** Four GWAS methods were used, Logistic Regression (performed by PLINK), FarmCPU, BOLT-LMM, and BLINK. The East Asian lung cancer population included 8,807 samples; each sample was genotyped with 629,968 SNPs (filtered by Minor Allele Frequency > 0.05, leaving 444,758 SNPs for the association study). The names of lung cancer candidate genes (Qing et al., Nature Genetics, 44, 1330-1335, 2012) with significant SNPs nearby were labeled on the BLINK plot. The distances between significant SNPs and candidate genes were also labeled.
