# Supplementary material for: BLINK: a package for the next level of genome-wide association studies with both individuals and markers in the millions
Source: Gigascience. 2018 Dec 11;8(2):giy154. doi: 10.1093/gigascience/giy154 (PMC6365300; doi:10.1093/gigascience/giy154)
Supplement: Supplemental Files [file giy154_supplemental_files.zip › S6_Figure.docx]

**
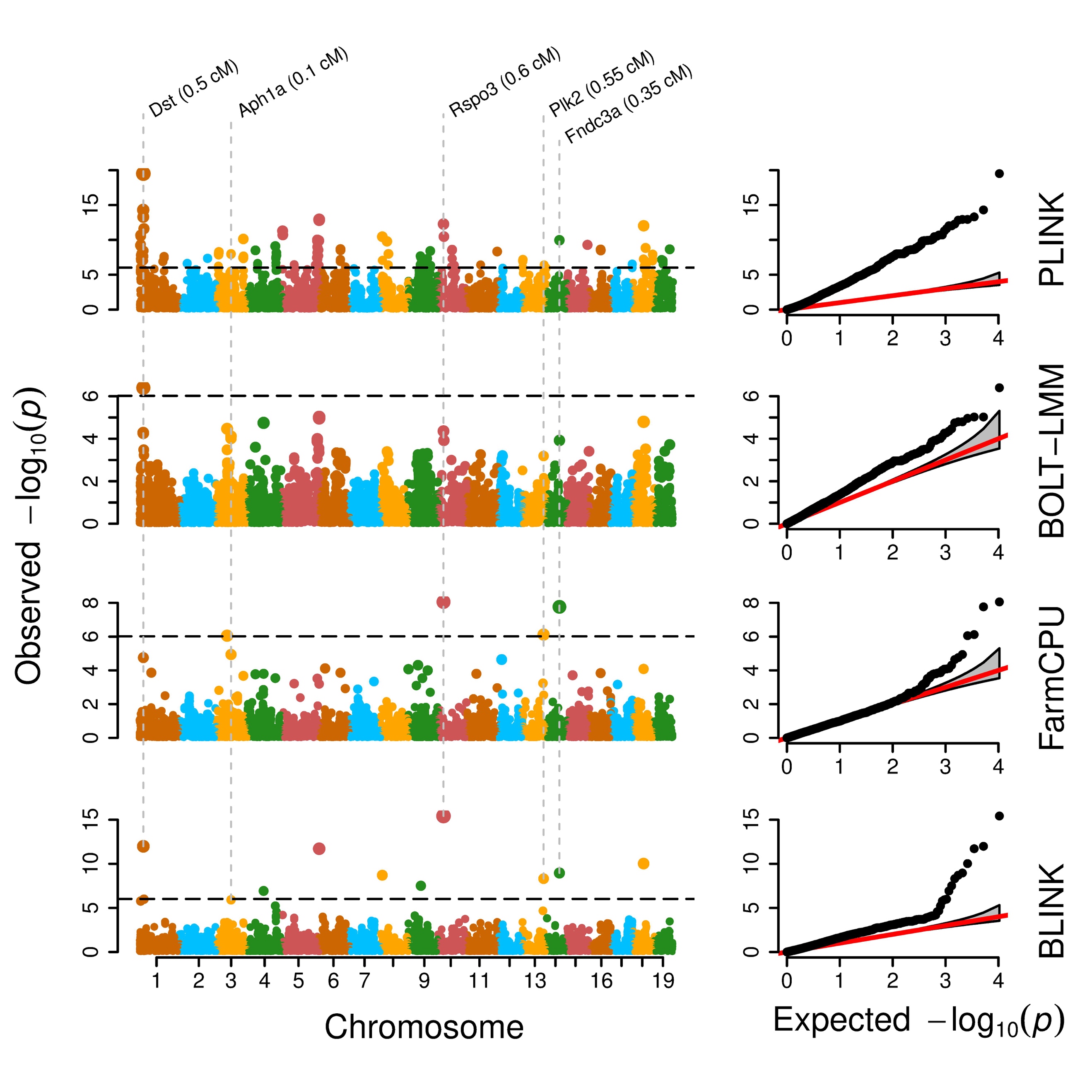
**

**S6 Fig. Association studies of weight growth intercept in mouse.** Four GWAS methods were used, GLM (performed by PLINK), FarmCPU, BOLT-LMM, and BLINK. The population included 1,940 samples; each sample was genotyped with 12,226 SNPs (filtered by Minor Allele Frequency > 0.05, leaving 10,432 SNPs for the association study). GLM included the first three PCs as covariates to control population structure. The names of weight growth intercept candidate genes and QTL with significant SNPs nearby were labeled on the BLINK plot. The distances between significant SNPs and candidate genes/QTL were also labeled. All QTLs’ information came from Mouse Genome Informatics (URL: <http://www.informatics.jax.org/>).
