# Supplementary material for: BLINK: a package for the next level of genome-wide association studies with both individuals and markers in the millions
Source: Gigascience. 2018 Dec 11;8(2):giy154. doi: 10.1093/gigascience/giy154 (PMC6365300; doi:10.1093/gigascience/giy154)
Supplement: Supplemental Files [file giy154_supplemental_files.zip › S7_Figure.docx]

**
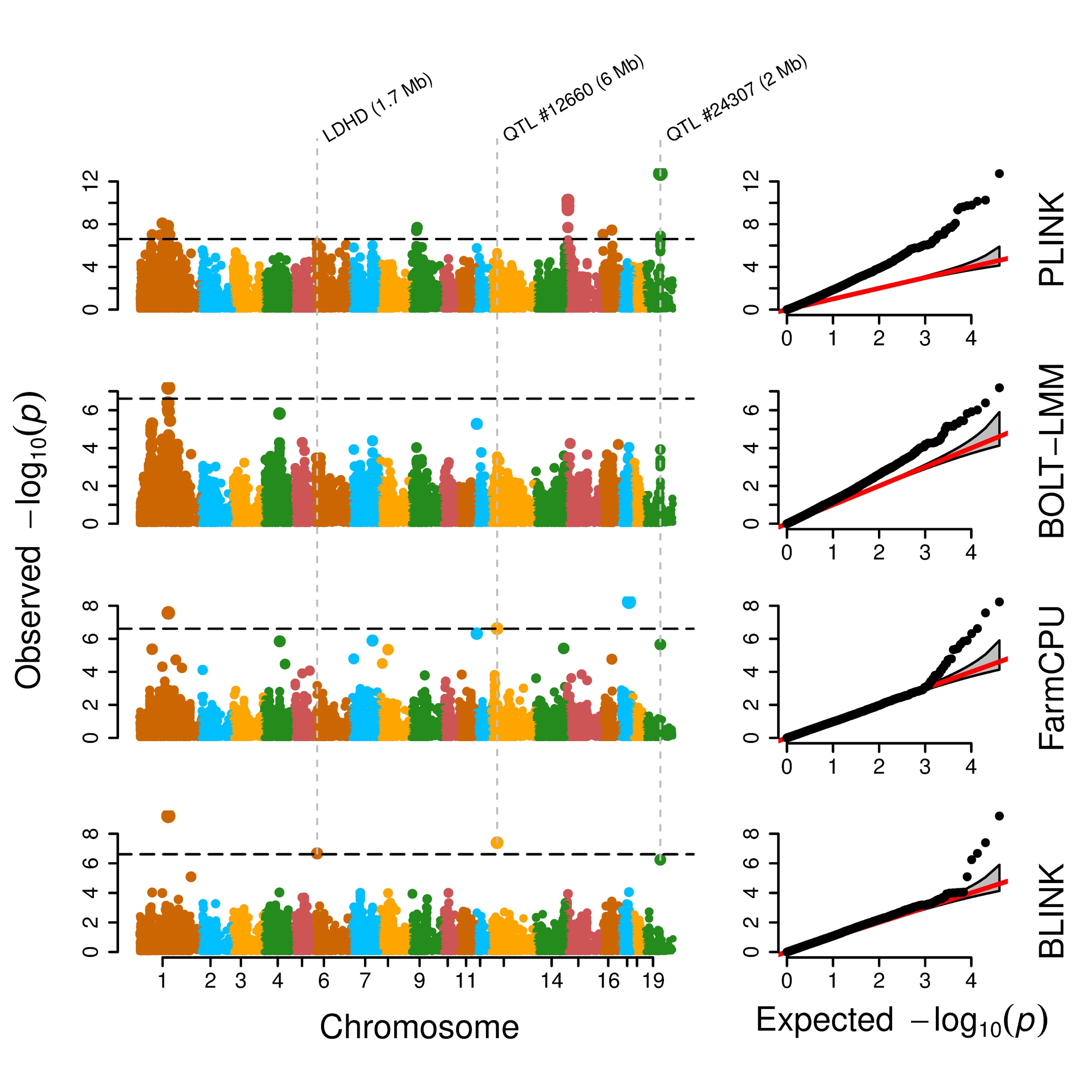
**

**S7 Fig. Association studies of last rib backfat thickness in pig.** Four GWAS methods were used, GLM (performed by PLINK), FarmCPU, BOLT-LMM, and BLINK. The population included 820 samples; each sample was genotyped with 64,212 SNPs (filtered by Minor Allele Frequency > 0.05, leaving 40,748 SNPs for the association study). GLM included the first three PCs as covariates to control population structure. The names of backfat thickness candidate genes and QTL with significant SNPs nearby were labeled in the BLINK plot. The distances between significant SNPs and candidate genes/QTL were also labeled. All QTLs’ information came from the Pig Quantitative Trait Locus Database (PigQTLdb, URL: <http://www.animalgenome.org/cgi-bin/QTLdb/SS/index>).
