# Supplementary material for: BLINK: a package for the next level of genome-wide association studies with both individuals and markers in the millions
Source: Gigascience. 2018 Dec 11;8(2):giy154. doi: 10.1093/gigascience/giy154 (PMC6365300; doi:10.1093/gigascience/giy154)
Supplement: Supplemental Files [file giy154_supplemental_files.zip › Table_S1.docx]

**Table S1. Properties of real genotypes and parameters of phenotype simulation*.**

| Property | Human | Maize | Arabidopsis | Mouse | Pig |
| --- | --- | --- | --- | --- | --- |
| Sample size | 8,807 | 2,279 | 1,179 | 1,940 | 820 |
| No. markers | 629,968 | 681,258 | 214,545 | 12,226 | 64,212 |
| No. QTNs | 500 | 100 | 50 | 100 | 50 |
| Heritability | 0.75 | 0.75 | 0.75 | 0.75 | 0.75 |
| QTN effect distribution | Normal | Normal | Normal | Normal | Normal |

* Real genotypes were used from five species, including human, maize, Arabidopsis, mouse, and pig. Upon sample size, the different number of markers were sampled as quantitative trait nucleotides (QTNs). For the largest dataset from the human with 8,807 individuals, 500 QTNs were used to simulate the phenotype. For the dataset with individuals around one thousand (Arabidopsis and pig), 50 QTNs were used. The other two datasets contain about two thousand individuals, and 100 QTNs were used. The simulated phenotypes have a heritability of 0.75, and the effects of the QTNs follow a normal distribution for all the species.
