# Supplementary material for: BLINK: a package for the next level of genome-wide association studies with both individuals and markers in the millions
Source: Gigascience. 2018 Dec 11;8(2):giy154. doi: 10.1093/gigascience/giy154 (PMC6365300; doi:10.1093/gigascience/giy154)
Supplement: Supplemental Files [file giy154_supplemental_files.zip › Table_S2.docx]

**Table S2. The information of** **operating system and machine configuration of computers for computing speed evaluation.**

| Machine ID | Machine configration | Operating system |
| --- | --- | --- |
| Mac 1 | 3.1 GHz dual-core Intel Core i5 | Sierra 10.12 |
| Mac 2 | 2.6 GHz quad-core Intel Core i7 | Sierra 10.12 |
| Mac 3 | 3.0 GHz 8-Core Intel Xeon E5 | Sierra 10.12 |
| Mac 4 | 2.7 GHz 12-Core Intel Xeon E5 | Sierra 10.12 |
| Linux 1 | 2.2 GHz dual-core Intel Core 2 | Ubuntu 13.10 |
| Linux 2 | 2.5 GHz 8-Core Intel Xeon E5 | Ubuntu 15.04 |
| Linux 3 | 2.3 GHz 10-Core Intel Xeon E5 | CentOS release 7.2.1511 |
