# Supplementary material for: BLINK: a package for the next level of genome-wide association studies with both individuals and markers in the millions
Source: Gigascience. 2018 Dec 11;8(2):giy154. doi: 10.1093/gigascience/giy154 (PMC6365300; doi:10.1093/gigascience/giy154)
Supplement: Supplemental Files [file giy154_supplemental_files.zip › Table_S3.docx]

**Table S3. The comparison of command lines between BLINK and PLINK.**

| Function | BLINK | PLINK |
| --- | --- | --- |
| Input | **--file [fileName]** **--[fileFormat]** | **--bfile [fileNmae]**  **--file [fileName]** |
| Traits selection | **--trait [trait number]** | **--pheno [additional fileName] –mpheno [trait number]** |
| GWAS | **--gwas** | **--****assoc**  **--linear** |
| Parallel computing | **--parallel [0/1]** | **--threads [number of threads]** |
