# Supplementary figures and images for: BLINK: a package for the next level of genome-wide association studies with both individuals and markers in the millions
Source: Gigascience. 2018 Dec 11;8(2):giy154. doi: 10.1093/gigascience/giy154 (PMC6365300; doi:10.1093/gigascience/giy154)

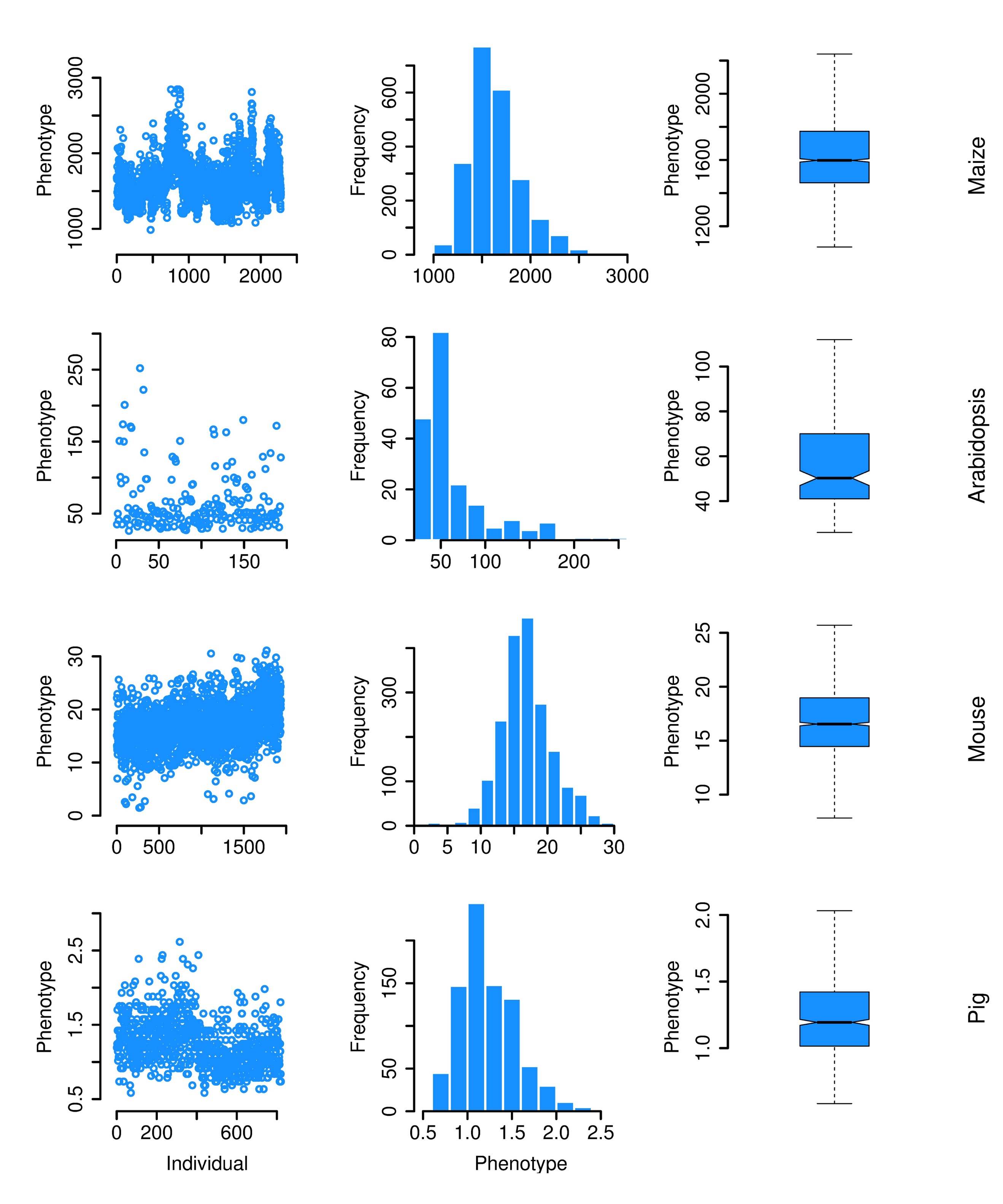


**S2 Fig. The distribution of real phenotypes data in maize, *Arabidopsis thaliana*, mouse and pig.**

Supplement: Supplemental Files [file giy154_supplemental_files.zip › S2_Figure.docx]
