# Supplementary material for: BLINK: a package for the next level of genome-wide association studies with both individuals and markers in the millions
Source: Gigascience. 2018 Dec 11;8(2):giy154. doi: 10.1093/gigascience/giy154 (PMC6365300; doi:10.1093/gigascience/giy154)
Supplement: Response_to_Reviewer_Comments_Revision_2.pdf [file giy154_response_to_reviewer_comments_revision_2.pdf]

Author's Response To Reviewer Comments

Close

Dear Sir/Madam

We have made the necessary corrections as suggested by Dr Scott Edmunds - See Version 26-11-2018.

Kind Regards

Dr Hassan Rahmoune

Close
